# Kinship practices in the early state El Argar society from Bronze Age Iberia

**DOI:** 10.1038/s41598-022-25975-9

**Published:** 2022-12-27

**Authors:** Vanessa Villalba-Mouco, Camila Oliart, Cristina Rihuete-Herrada, Adam B. Rohrlach, María Inés Fregeiro, Ainash Childebayeva, Harald Ringbauer, Iñigo Olalde, Eva Celdrán Beltrán, Catherine Puello-Mora, Miguel Valério, Johannes Krause, Vicente Lull, Rafael Micó, Roberto Risch, Wolfgang Haak

**Affiliations:** 1 Department of Archaeogenetics, Max Planck Institute for Evolutionary Anthropology, 04103 Leipzig, Germany; 2 Instituto Universitario de Investigación en Ciencias Ambientales de Aragón, IUCA-Aragosaurus, Zaragoza, Spain; 3 Department of Prehistory, Universitat Autònoma de Barcelona, Barcelona, Spain; 4 School of Mathematical Sciences, University of Adelaide, Adelaide, 5005 Australia; 5 Independent researcher, Murcia, Spain; 6 BIOMICs Research Group, Department of Zoology and Animal Cell Biology, University of the Basque Country UPV/EHU, Vitoria-Gasteiz, Spain; 7 Ikerbasque-Basque Foundation of Science, Bilbao, Spain; 8 Department of Genetics, Harvard Medical School, Boston, MA USA

**Keywords:** Genetics, Archaeology, Anthropology, Biological anthropology, Social anthropology

## Abstract

The Early Bronze Age in Europe is characterized by social and genetic transformations, starting in the early 3rd millennium BCE. New settlement and funerary structures, artifacts and techniques indicate times of change with increasing economic asymmetries and political hierarchization. Technological advances in metallurgy also played an important role, facilitating trade and exchange networks, which became tangible in higher levels of mobility and connectedness. Archeogenetic studies have revealed a substantial transformation of the genetic ancestry around this time, ultimately linked to the expansion of steppe- and forest steppe pastoralists from Eastern Europe. Evidence for emerging infectious diseases such as *Yersinia pestis* adds further complexity to these tumultuous and transformative times. The El Argar complex in southern Iberia marks the genetic turnover in southwestern Europe ~ 2200 BCE that accompanies profound changes in the socio-economic structure of the region. To answer the question of who was buried in the emblematic double burials of the El Argar site La Almoloya, we integrated results from biological relatedness analyses and archaeological funerary contexts and refined radiocarbon-based chronologies from 68 individuals. We find that the El Argar society was virilocally and patrilineally organized and practiced reciprocal female exogamy, supported by pedigrees that extend up to five generations along the paternal line. Synchronously dated adult males and females from double tombs were found to be unrelated mating partners, whereby the incoming females reflect socio-political alliances among El Argar groups. In three cases these unions had common offspring, while paternal half-siblings also indicate serial monogamy or polygyny.

## Introduction

The beginning of the European Bronze Age (BA) involved drastic social changes that resulted in strong political centralization, growing economic inequality, and settlement and demographic disruptions. These social changes are striking in particular regions, such as Central Europe, Brittany, southern England and southeastern Iberia, where the unequal distribution of wealth, as reflected in the grave goods, becomes more apparent and consistent^1–3^.

Recent genomic analyses have suggested that these changes were related to the westward expansion of "steppe-related ancestry" and the reduction in diversity of male lineages in most of Europe, following a process that began in the early 3rd millennium cal BCE in Eastern Europe^4–8^. In this context, increased violence and social coercion could have played a role in new social relations (e.g.,^9–12^). However, the role and nature of population movements (violent or peaceful), or expansions in this process remain a matter of debate. Following the introduction of game-shifting innovations^13^, other factors, such as new economies, climate change or infectious diseases^14–17^ might also account for socio-economic and genetic changes detected throughout the transformative times of the 3rd millennium BCE in Europe. In addition, the magnitude of transformations varied in each region. More integrative work is needed to understand the relative contribution of different factors in such intricate processes of change at local and cross-regional levels.

**Figure 1:**
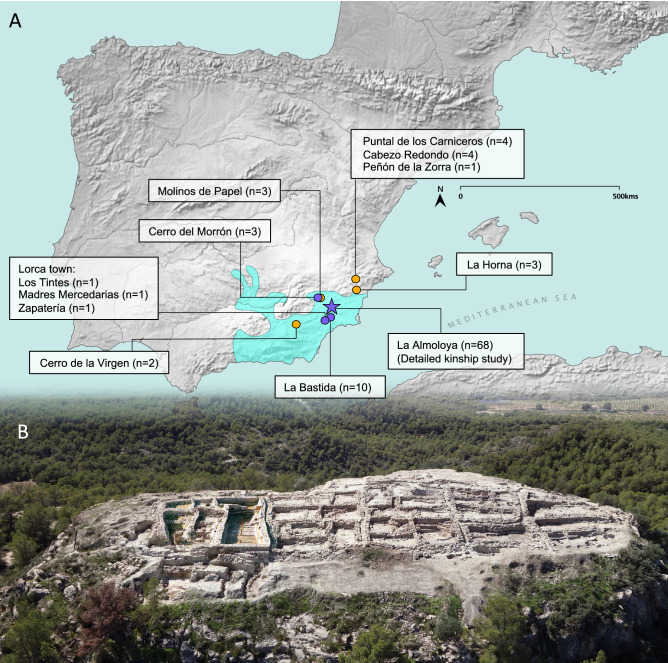
Early bronze age El Argar in southern Iberia. (**A**) Map of Iberia and location of the Argaric and other Bronze Age nearby sites. The map was created using QGIS 3.12 (https://qgis.org/en/site/) and uses Natural Earth vector map data from (https://www.naturalearthdata.com/downloads/). (**B**) A view of the La Almoloya hilltop site from the East.

The archeological complex of El Argar in southeastern Iberia provides a key case study for deepening our knowledge of social-political reorganization in the European Early BA (EBA) (Fig. 1A). El Argar is one of the archeological entities in which a socio-economic divide^18,19^ and a genetic shift are clearly documented^8^, and it is arguably one of the first highly complex societies in Western Europe to reach the status of an early state^1,19–21^. El Argar developed over three phases from ca. 2200 to 1550 cal BCE^19^, spreading from its heartland in the coastal lowlands into the inner highlands, and covering ~ 35,000 km^2^ at its peak. The Argaric archeological record includes permanent and densely populated hilltop settlements of up to 5 hectares in size. These settlements were organized and managed hierarchically, with evidence of public buildings for political decision-making^22^, and structures for water supply. Moreover, large-scale storage and processing of cereal crops^23^, specialized pottery and metallurgical production, and intensive subsistence systems combining rainfed agriculture, manuring and small-scale irrigation are found at Argaric sites^18,19,24^.

Argaric sites offer a unique opportunity to address questions of biological relatedness and kinship, since a substantial proportion of the population was buried in single or double tombs, placed under the settled areas. These funerary practices allow us to link individuals, grave goods, tombs, and architectural units diachronically, which is rare in late prehistoric Europe. To date, Argaric archeology has approached questions of kinship through the analysis of double tombs, which represent nearly 20% of burials in certain sites. On average, more than half of these tombs were assigned to two adults, a quarter were occupied by an adult and a child, and the combination of two children was far less common (~ 10%)^25^. The main focus of previous research has been on double tombs with two adults, usually a male and a female, providing the basis for the hegemonic model since the late nineteenth century: specifically, that these graves were thought to reflect heterosexual, monogamous couples (‘marriages’) as the basis for nuclear families^26–29^.

To test this ‘marriage’ hypothesis, male and female skeletons in double tombs were sampled for radiocarbon dating in the late 1980s. A statistical analysis of paired ^14^C dates from a sample of 23 double burials suggested a cross-generational gap for most pairs of individuals^30^, which led to the alternative hypothesis that the relationships between adults in double tombs were genealogical rather than socio-political. As a result, descent/consanguinity, matrilocality and matrilineality were proposed as the main principles of Argaric kinship practices^25,31–33^.

The aim of this study is to use state-of-the-art ancient DNA methods to determine the nature of the genetic relationships between individuals from El Argar contexts, and to use these as new evidence to shed light on kinship practices and social organization of the Argaric society, including inheritance rules that are potentially linked to households. Moreover, we aim to investigate the prevailing hypotheses on Argaric kinship practices, through an interdisciplinary approach that combines genetic, osteological, and fine-grained contextual information from archeological excavations. The bulk of our data comes from the site of La Almoloya in Murcia^22,34^, which, due to its unique preservation and extensive excavations, features a variety of burial types and grave goods, complemented by detailed anthropological data and a radiocarbon and stratigraphy-based chronology (Supplementary 1, Fig. 1B). The results are expected to improve our understanding of one of the earliest, highly complex societies in BA Europe.

## Data overview and detection of genetic relationships

At La Almoloya we sampled 86 individuals with suitable morphological preservation from a total of 101 graves, containing the remains of 128 individuals (Dataset S1.1). We obtained high-quality genome-wide data (1240 k SNP capture data) for 68 individuals (79% success rate) passing our quality control thresholds (< 4% contamination, characteristic aDNA damage profiles, unambiguous sex determination) (“Materials and methods”, Dataset S1.2). The 68 individuals from La Almoloya cover El Argar phase 2 (n = 41) and phase 3 (n = 27) of the local stratigraphy (2000–1750 cal BCE and 1750–1550 cal BCE, respectively), and we observed no bias with respect to post-depositional or taphonomic factors (“Materials and methods”). The relative frequencies of females and males, adults and subadults with genetic results match those of the physical anthropological examination for each of these demographic groups, indicating that the sample is representative of the group buried at the site (Dataset S1.3).

Another three individuals from La Almoloya yielded autosomal SNP data below the threshold of 20,000 SNPs: ALM037 (18,226 SNPs), ALM045 (10,970 SNPs), ALM033 (356 SNPs) with no contamination estimate (Dataset S1.2). These three individuals were only included in some analyses to confirm or exclude potential 1st-degree biological relationships, which is also possible for low coverage data (Supplementary 2). To estimate the biological relatedness among La Almoloya individuals and also among and between other published BA individuals from Iberia, we first calculated the pairwise mismatch rate (PMR) (Supplementary 2, Fig. S1), which also provides a general threshold of background (un-)relatedness in BA Iberia from randomly drawn pairs of individuals^6,7,35^. Here, individuals from neighboring El Argar and Iberian BA groups were of particular interest for the comparison and discussion of results (Dataset S1.2^8^).

**Figure 2:**
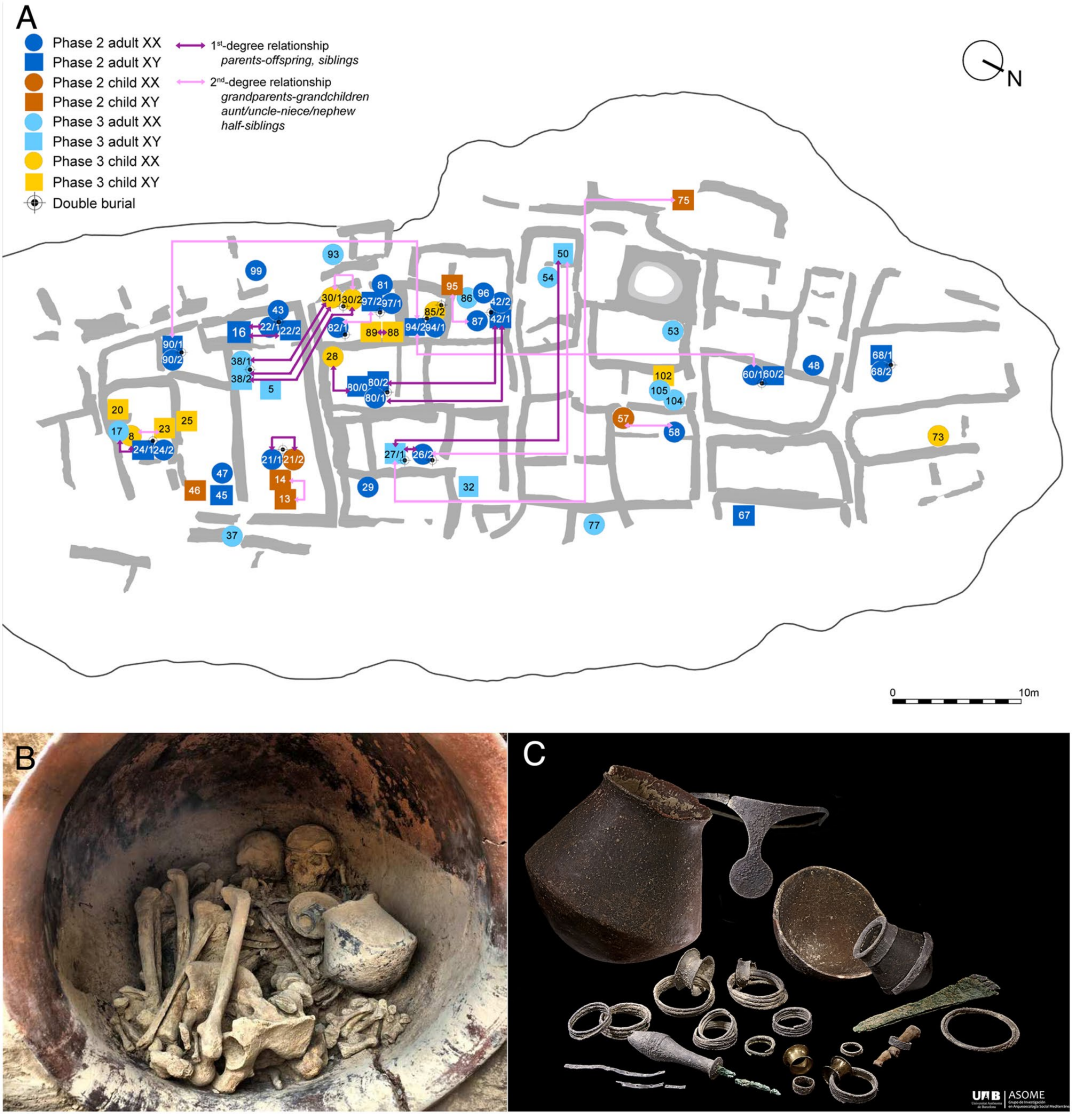
(**A**) Plan of La Almoloya phase 3 highlighting the position of burials that have yielded sufficient aDNA and close genetic relatedness between individuals up to the 2nd degree. For full pedigrees see SI Appendix, Sect. 3. (**B**) Exemplary *pithos* double burial and grave goods (**C**) from La Almoloya burial AY38.

**Figure 3:**
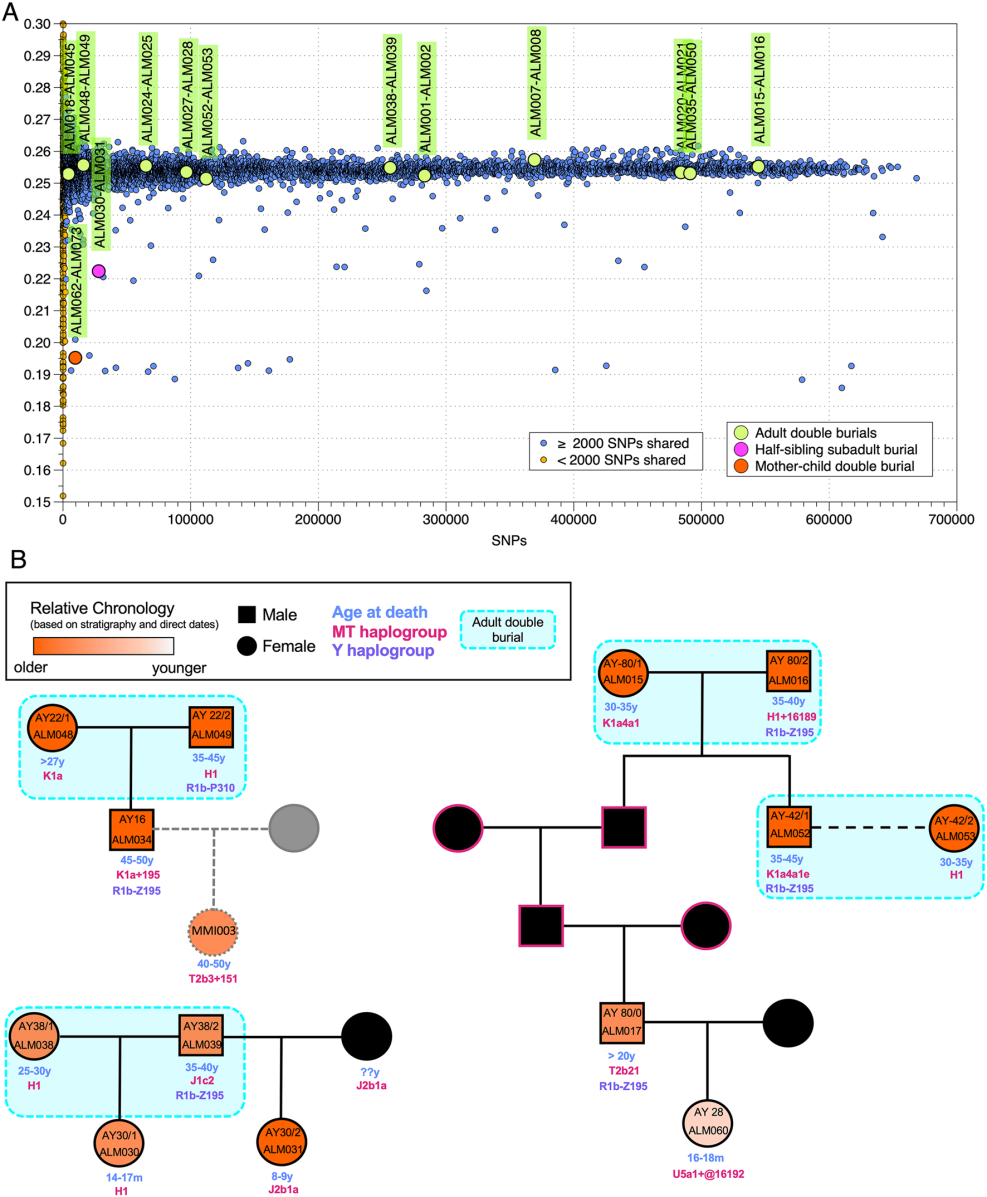
Summary of the genetic results from the double burials at Almoloya. (**A**) Results of the Pairwise-mismatch rate (PMR) analysis including all pairs of individuals from the Iberian Bronze Age available to date. The x-axis shows the number of overlapping SNPs between each pair and the y-axis the coefficient of relatedness. All adult double burials fall within the range of randomly drawn pairs of individuals from the Iberian Bronze Age. (**B**) Reconstructed pedigrees of three cases involving adult double burials and their common offspring. Below the colored squares and circles is the contextual information of all adult double tombs, including sex, age at death, mitochondrial and Y-chromosomal haplogroups, direct ^14^C dates, as well as stratigraphy. Pink outlines reflect interchangeable genetic sexes and gray dashed lines indicate pedigrees reconstructed from low coverage data.

In total, we report 13 1st-degree-relationships and 10 2nd-degree-relationships among the 68 individuals at La Almoloya, involving 34 individuals (50%) for which genome-wide data was generated (Fig. 2, Supplementary 2; Fig. S1–S4*,* Dataset S1.4-S1.6). Of note, 1st-degree relatives can be parent–offspring or full siblings, and 2nd-degree relationships include aunt-uncle/niece-nephew, grandparent-grandchild, and half-siblings (who share only one of the biological parents). We were able to reconstruct seven pedigrees involving all 1st-degree relationships and to extend some of these pedigrees up to five generations (Fig. 3, Supplementary 3, Fig. S5). In addition, we found pairs that were 2nd-degree relatives. We set these aside as ‘unresolved pedigrees’ due to the lack of bridging 1st-degree relatives and the fact that we were unable to unambiguously determine the exact genealogical relationships despite the integration of osteological and archeological data (Supplementary 3, Dataset S1.4–S1.6). With the help of an Identity-by-descent (IBD) analysis of imputed high-quality genome-wide data from selected pairs (> 600,000 SNPs; “Materials and methods”), we also found evidence for more distant genetic relationships up to the 6–7th degree between individuals from three of the seven reconstructed pedigrees, as well as more distant connections between sites (Supplementary 2, Fig. S4, Dataset S1.7). Reconstructed pedigrees from nearby BA sites are shown in Supplementary 4.

We notice a similar frequency of 1st-(N = 13) and 2nd-degree (N = 10) related individuals. As the number of 2nd-degree relatives is expected to double that of the 1st-degree relatives, the burials at La Almoloya seem to emphasize the closest biological ties if we disregard the number of unrelated individuals also buried there. While the number of samples is too small to provide robust statistical support, the underrepresentation of 2nd-degree relatives indicates that not all biological relatives were buried in the settlement, which suggests inter-site mobility, involving residential changes for a substantial part of the population. A concomitant increase in child burials during phase 3 (a common feature during late Argaric times ^30^), suggests an emphasis in funerary rites on the closest biological offspring, or an increase in child mortality.

In what follows, we describe the results of the estimation of biological relatedness in reference to the physical distances between the burials, starting with closely inhumated individuals in double burials followed by relatives separated by larger distances at the site.

### Double burials of two adults

Double burials are an iconic feature of the El Argar group and play a central role in the discussion of Argaric kinship. This type of burial had already been attested to in earlier times at southeast Iberian sites such as Molinos de Papel^36,37^, and continued to be practiced during phases 2 and 3 of El Argar. At La Almoloya, 48 out of 126 individuals (38%) were buried in 24 double tombs. The majority of these tombs (N = 20) contained two adults (fourteen from phase 2 and six from phase 3), while the rest were either two children or one adult and one child. In one of the adult double burials (AY80), the remains of a skull of a third individual were placed under a slab outside the cist, after the tomb had already been sealed.

We successfully retrieved genomic data from both skeletons for ten adult double burials (20 individuals). The genetic results confirmed the anthropological morphological sex determination of the individuals, and in all ten cases the double burials contained a male and a female, which were found to be genetically unrelated, with the coefficient of relatedness between them being 0.001 (ranging from − 0.024 to 0.022) (Fig. 3A, Dataset S1.4, Supplementary 2). Each pair of double burial adults yielded PMR values which were close to the baseline median of the PMR values, which was established from all unrelated pairs of Iberian BA individuals across many sites. Significant overlap of the calibrated ranges from direct radiocarbon dates of all analyzed pairs makes the coexistence of both individuals possible (Fig. 2, Supplementary 5, Fig S6, Dataset S1.2, Dataset S1.8). All analyzed adult couples carried different mitochondrial haplogroups, which ruled out direct maternal links (Dataset S1.2). This observation can also be extended to other Argaric sites, such as Cerro del Morrón, from where we also analyzed a contemporaneous, adult double burial that contained an unrelated female (CMO001) and male (CMO002), and other BA sites from southeastern Iberian BA, such as Molinos de Papel (MPD002, female and MPD003, male) (Supplementary 2*,* Dataset S1.2). In another adult double burial (AY82) we obtained good coverage data for the female individual (ALM018), but not for the male (ALM045), however the results of the biological relatedness tests still suggested that the pair was not related (Fig. 3A, Dataset S1.4). In addition, in three out of ten cases from La Almoloya, males and females from double burials had offspring together, and thus represented mates (Fig. 3B). This observation suggests the existence of female–male socio-political alliances in life, which were also symbolized in the funerary practices of the El Argar society. So far, the contemporaneous intra-site genomic studies have reported the presence of sexual partners at the site confirmed by common offspring but buried in different graves ^38^. In other examples, only one of the biological parents was found buried at the site ^39–41^.

An outstanding example is the high-status *pithos* double burial AY38 from the palatial building (Fig. 2B), in which a man (ALM039) and a woman (ALM038) were endowed with rich grave goods (Fig. 2C) and thus were interpreted as having been prominent members of the ruling class^22^. The woman was buried with one of the five silver diadems found uniquely at Argaric sites (the other four are from the eponymous El Argar site, about 100 km south of La Almoloya). This outstanding funerary item has been interpreted as a symbol of distinction and power of some El Argar women^22^. From the funerary context we know that the man died first, just shortly before the woman, because his skeleton was found beneath her featuring a remarkably low degree of disarticulation and joint displacement, in particular along the spine and the thorax. We found a common daughter in the double grave AY30 (ALM030) buried in a pit without goods in a different architectural complex, further away from her parents (Fig. 3B). ALM030 had passed away prematurely at an estimated age of 14–17 months.

Another outstanding example of a double burial containing partners is AY80. The stone cist contained a 30–35-year-old female (ALM015) and a 35–40-year-old male (ALM016) attributed to the high social class (Fig. 3B). The funerary context suggests that the woman had died first and her bones were then collected and deposited in a bundle on top of the male burial, which took place at a later time. Both had a common son (ALM052), who reached 45–50 years of age and was buried without grave goods 11 m away, together with the adult female ALM053, who was buried after him, both in another adult double burial AY42.

The skull of an adult male (ALM017) from outside the cist in which the couple from AY80 had been buried, was found to be related in the 3rd degree to both individuals in AY80. Additional IBD analysis of the pair ALM015 and ALM017 confirmed the 3rd-degree relationship in direct generational succession, who together with ALM016 thus represent great-grandparents and great-grandson (Fig. 3B; Supplementary 2*,* Dataset S1.7). In addition, we found that the great-grandson, ALM017, was 1st-degree related to ALM060, his 18–20-month-old daughter who was buried in a *pithos* in the same area. This constitutes the largest pedigree reconstructed in La Almoloya, which connects relatives over five generations spanning phases 2 and 3, from the great-great-grandparents (ALM015 and ALM016) to the great-great-granddaughter (ALM060), in a relatively close area, even though generations are missing, remained untyped, or might have been buried elsewhere (Fig. 3B, Supplementary 2, Fig. S4). While the small number of cases and limitations of ^14^C date ranges do not allow the generalized assumption of genealogical links across phases, this example argues against the possibility that the dramatic changes in settlement layout in phase 3 were undertaken by an entirely new group or dynasty. Genetic continuity at the population level between the two phases, as described in^8^, also rules out scenarios of drastic demographic changes (Supplementary 5, Fig. S7)*.*

Grave AY22 represents another adult double burial with common offspring (Fig. 3B). The burial contained a 35–45-year-old female (ALM048) and an adult male (ALM049), whose state of preservation did not permit a more precise age estimation. We identified their common adult son ALM034 in a single cist burial (AY16) less than 3 m away from AY22.

Taken together, males and females from adult double tombs were not found to be genetically related to each other. Irrespective of this finding, these individuals were involved in all 1st- and 2nd-degree relationships among adults at the site, which suggests that double burials displayed a central social role (Fig. 3B, Supplementary 2, Supplementary 3).

### Double burials with an adult and a child

At La Almoloya, we documented three double burials containing an adult and a subadult individual, of which only one could be analyzed genetically. Cist burial AY21 contained a 30–35-year-old woman (ALM073), who was buried holding a newborn female baby (ALM062) against the right side of her chest. As the skeletal superposition indicates, the synchronic inhumation of the two bodies and the genetic analysis suggests a mother/daughter relationship (Fig. S5A), where complications in the period after childbirth could be considered the likely cause of death. It is noteworthy that the child carried the aneuploid XXX-syndrome ^8^, but this condition is unlikely to have caused the untimely death of both the baby and her mother ^42^.

The combination of adult/subadult individuals in double burials (25%) is relatively uncommon at El Argar sites, and the example from tomb AY21 might hint at exceptional circumstances involving a close genetic relationship. This might be the case for tomb AY85, in which another female neonate, whose sex has been determined genetically (ALM079), was found in the arms of an adult female (ALM066) and in a position remarkably similar to tomb AY21. Unfortunately, the sample of the adult female did not produce sufficient genetic data and therefore we could not ascertain a putative mother/daughter relationship.

Conversely, tomb BA6 at La Bastida shows that other scenarios are possible. Here, a 25–30-year-old male (BAS002) was buried alongside a newborn boy (BAS026), but the two were not genetically related (Dataset S1.2). It is important to note that this burial cannot be considered simultaneous as in the other two cases, since part of the *pithos* rim broke when the tomb was reopened and the fragments ended up on top of the adult pelvis and below the neonate skeleton, thus providing evidence of successive inhumations. However, radiocarbon dates were not statistically different at the 95% confidence level (Supplementary 5). It is possible that this burial was meant to represent a father/son relationship, but whether the adult male buried in BA6 would be aware of the real biological fatherhood or not (as stepfather and therefore social kin) remains an open question.

### Double burials of children

We successfully recovered DNA from the only double burial at La Almoloya (AY30) with two children. The grave consisted of a small pit, in which a female toddler (ALM030) of 14–17 months was first buried, followed later by an 8- or 9-year-old girl (ALM031). There were no grave goods associated with ALM031, and from the funerary context we infer that she was buried after ALM030, as her skeleton was found fully articulated and partly on top of the jumbled post-cranium of the younger girl ALM030. We detected a half-sibling relationship between these two girls on their father’s side (Fig. 3, Supplementary 3), which indicated that the adult male ALM039 from the wealthiest grave AY38 of La Almoloya, located in the nearby palatial building, was the father of both. It is noteworthy that the adult female ALM038, buried alongside this man, was the mother of only one of the girls (ALM030), and that we have not identified the biological mother of ALM031 among the successfully typed individuals. The archaeological context does not provide clues as to whether the two mothers lived at the same time or not, nor whether this case represents an example of serial monogamy, or, alternatively, polygamy. However, the fact that the half-sisters were entombed together reflects awareness (on the part of the people who buried them) of the kin relationship between the two children, irrespective of their different biological mothers, and very likely also the acknowledgement of fatherhood on behalf of ALM039. However, it is also possible that these unions were temporal and dissolvable.

The instances in which siblings were identified genetically also represent variable archaeological contexts and situations open to interpretation. We also detected two siblings buried together in tomb BA23 from La Bastida, which, like AY30, also dates to the late Argaric phase (Supplementary 4). A 9-to-11-month-old girl BAS017 was buried in a ceramic vessel, followed by her brother BAS018, who died shortly after at roughly the same age. Conversely, in another case of siblings from La Almoloya, two boys (ALM080 and ALM081) were buried very close to each other but in separate graves (AY88 and AY89, respectively) (Fig. S5).

As a result, we observe that double tombs containing at least one child feature close biological relationships, except for the case of the adult and newborn male found at La Bastida (BA6), which deviates from the double burials of adults.

### Genetic relationships beyond double tombs

We also observed several biological relationships between individuals buried in single tombs and therefore explored the degrees of relatedness in the light of chronological and spatial distances. Phase 3 at La Almoloya is characterized by a network of housing complexes that were built around 1750 cal BCE, and which form a large part of the structures visible today (Fig. 2A). However, the layout of the preceding phase 2 was largely dismantled by the urbanization efforts of phase 3. Thus, building complexes cannot be used as a general background, and it is advisable to operate with raw distances only.

To analyze the spatial distribution of biologically related individuals, we plotted the physical distances between all 1st- and 2nd-degree related pairs of individuals (Fig. 2A). We find that most of the individuals with close parental links were buried less than 5 m apart from each other. Interestingly, half-siblings (biologically 2nd-degree relatives) are spatially as close as full siblings (1st-degree relatives), while all other 2nd-degree related individuals (uncle-aunt/nephew-niece and grandparent-grandchild relationships) are separated by 19–32 m, and are often buried under the floors of different buildings of phase 3. We documented a case of full siblings (ALM080 and ALM081) located in separate but close *pithoi* graves without grave goods. Both male individuals died prematurely, one at the age of 14–20 months (ALM080) and the other at the age of 18–24 months (ALM081), and while the time elapsed between their deaths remains unknown, this situation suggests the intentional placing of the two brothers in a nearby space (Fig. S5). As mentioned above, other full siblings from La Bastida (genetically male and female) were found buried together in tomb BA23 (Supplementary 4). These two examples may reflect social ties among subadult siblings regardless of their biological sex.

We detected a possible half-sibling relationship on the father’s side between ALM068 (a 14–16-month-old female baby from tomb AY8) and ALM078 (a 14–16-month-old male from tomb AY23 interred sometime later), buried in separate graves but within the same housing complex. The mother of ALM068 was buried in a separate tomb (AY17-ALM077), close to her daughter but not to the boy (ALM078), to whom she is biologically unrelated. Another possible, but perhaps less plausible interpretation for the 2nd-degree relationship between the two children is that the boy (ALM078) was the uncle of the girl (ALM068) on her father’s side (albeit one generation younger), as he is not related to her mother, ALM077 (Fig. S5).

Another half-sibling relationship on the father’s side can be suggested for ALM046 (a 14–18-month-old boy), buried in grave AY13, and ALM047 (a 6–7-month-old male infant) buried in grave AY14, none of which had grave goods. In this case, we found neither the father nor the mother among the successfully genotyped individuals, and we cannot completely rule out the possibility of an uncle/nephew relationship. However, these individuals were buried close to one another, as was also the case with other pairs of siblings, which strongly hints at specific social and familial ties (Supplementary 3).

In summary, an aunt-uncle/niece-nephew relationship cannot be excluded in cases in which two children are related in the 2nd degree. It is worthwhile noting that half-siblings, which would in every case be linked via the paternal line, make polygamy (polygyny) and serial monogamy plausible practices to consider. However, we also found other 2nd-degree relationships between an adult and a subadult for which we cannot discard an avuncular relation, such as ALM004 (adult female) and ALM075 (infant female), or ALM019 (adult female) and ALM069 (infant male) (Supplementary 3).

Lastly, we were able to infer more distant relatives by calculating the number and fraction of segments in the 1240 k SNP data that are identical-by-descent (IBD) in individuals with > 600,000 SNPs after imputation (“Materials and methods”, Supplementary 2). This method not only allowed us to confirm and distinguish between close 1st- and 2nd-degree relationships but also to estimate biological relatedness up to the 6th–7th degree, by which we were able to connect three of the seven main pedigrees reconstructed at La Almoloya (Dataset S1.7, Supplementary 3).

### Inferring kinship practices

**Figure 4:**
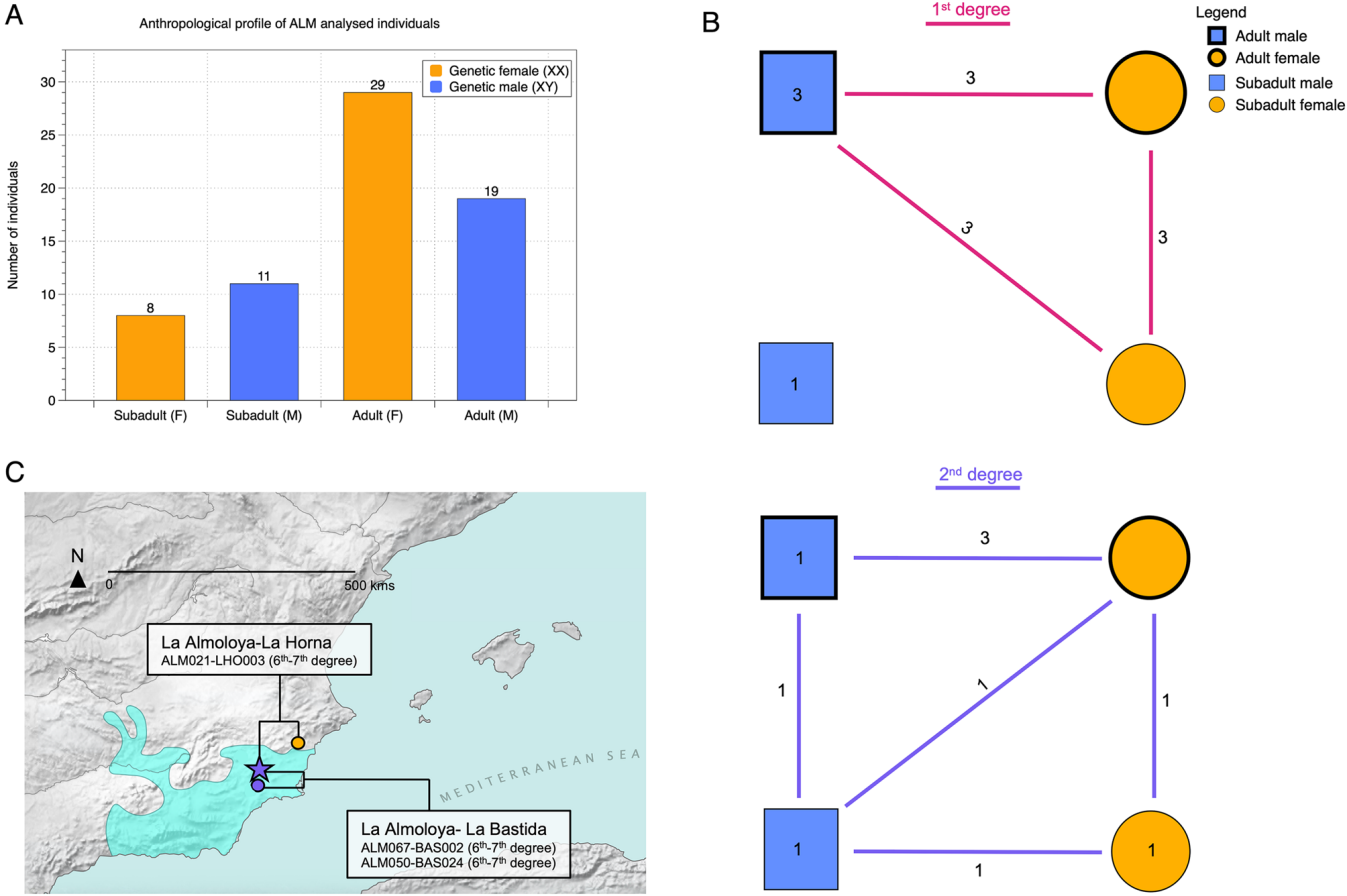
Overview of genetic sex and age at death determinations and total numbers of observed relationships per category and long-distance relatives outside La Almoloya. (**A**) Simplified age classes of the individuals analyzed from La Almoloya: subadult (including infant (0–3 years) and child (3–12 years)) and adult (including young adult (20–35 years), middle adult (35–50 years), and old adult (50+ years)^56^) females and males. Of note, adolescent individuals (12–20 years) were not found at the site; (**B**) total number of 1st- (pink) and 2nd-degree (purple) relatives between age and sex classes as summarized in (**A**). Numbers reflect the number of links in the pedigrees between age/sex classes and numbers inside symbols reflect the number of links within each class. The graph illustrates the absence of either 1st- or 2nd-degree relationships among adult females; (**C**) Long-distance relatives (6–7th degree) as indicated by shared IBD-blocks between individuals from La Almoloya and other BA archaeological sites. The map was created using QGIS 3.12 (https://qgis.org/en/site/) and uses Natural Earth vector map data from (https://www.naturalearthdata.com/downloads/).

Following the individual-based evaluation of biological relatedness at La Almoloya, we compared the structure of the reconstructed pedigrees from all related individuals and looked for links across generations that would signal lineality or inheritance along preferential parental lines. Here, we observed that all reconstructed pedigrees are linked through the paternal side and, in one case, the male lineage can be traced over at least five generations (Fig. 3, Supplementary 3). In addition, in all other cases of 2nd- and 3rd-degree relationships (six 2nd-degree pairs), for which the pedigree could not be fully established or extended due to missing 1st-degree related individuals, we observe that the possible alternative pedigrees can also only be explained through the paternal line (Supplementary 3). All 1st-degree relationships among adults involved at least one adult male (3 out of 19 adult males analyzed) (Fig. 4). No adult male has an adult daughter, sister, brother, or adult half-siblings also buried at the site. The few 2nd-degree relationships involving adults were all also between males (4 out of 19 adult males analyzed) (Fig. 4).

Conversely, we found no 1st- or 2nd-degree relationships between adult women (0 out of 30 adult women analyzed). Women related in the 1st degree at the site were mothers of either girls (AY21/2-ALM062, AY30/1-ALM0030, AY8-ALM068) or adult males (AY16-ALM034, AY27/1-ALM058 and AY42/1-ALM052), but not boys. These women were not related to any other adult women (Fig. 4) and had no parents at the site. The same holds true for 2nd-degree related females, then considered integrated lineage females, who were found to be aunts and/or grandmothers (AY58-ALM004, AY87-ALM019, AY26/2-ALM086) of both girls and boys, but, again, never related to adult women (Fig. 4).

These results obtained by reconstructing the pedigrees are in line with results that have already been reported at the meta-level in^8^, where it was described that males of La Almoloya had more close relatives at the site than females. This observation was made based on significantly higher observed *f*_3_-statistics for *f*_*3*_ *(male, male; Mbuti)* than *f*_*3*_ *(female, male; Mbuti)* than *f*_*3*_ *(female, female; Mbuti)* (after excluding all 1st- and 2nd-degree related pairs). Although the *f*_*3*_*-outgroup* statistic is used at the population level to measure shared genetic drift between two populations after the split from a common outgroup, it has been also useful to identify 1st-degree relatives as they share half of their genomes and thus, will report higher *f*_3_-values^4,8^.

Examining the paternal lineages, we observe a higher residential stability, not only at the site but also in some specific housing complexes (i.e., close proximity) (Fig. 2A). A prime example is ALM034/AY16, an adult male buried close to his parents, ALM048/AY22-1 and ALM049/AY22-2. A similar scenario could be inferred for female ALM086/AY26-2, buried in an adult double burial close to her adult son ALM058/AY27-1. Unfortunately, we did not obtain sufficient aDNA from the partner buried with ALM086/AY26-1 to prove the biological fatherhood between him and ALM058/AY27-1 (Supplementary 3, Fig. S5). Finally, another example of cross-generational paternal lineages buried closer in space are ALM015/AY80-1 and ALM016/AY80-2, an adult double burial, which also contained the skull of their adult great-grandson ALM017/AY80-0 (Fig. 3, Supplementary 3).

Other sources of information to infer lineality and locality can be gleaned from uniparentally inherited markers, such as mitochondrial DNA (mtDNA) and Y-chromosomal haplogroups. Irrespective of the varying genetic resolution, the finding of only one single Y-chromosome lineage in La Almoloya (R1b-P312 > Z195), which is also the predominant lineage across Iberia, is remarkable, but cannot be used to resolve relationship patterns at an intra-site level and thus suggests a much more common practice at a broader scale or a small/non-diverse source population of Y chromosome diversity. The diversity of the mtDNA is also similar to the diversity observed in Iberia in the preceding periods ^43^, with the exception of one female individual carrying mtDNA haplogroup R0a, which has not been reported to date from Iberia (Dataset S1.2).

Aside from close relationships within La Almoloya, we also found a close relationship between ALM034/AY16 and a 40–50-year-old female from Lorca, buried in Madres Mercedarias Tomb 4_1 (MMI003), another Argaric site about 50 km away. Due to the low coverage of MMI003, the biological relatedness tests returned an intermediate value between 1st- and 2nd-degree and thus need to be interpreted with caution. In the case of a 1st-degree relationship, MMI003 would be a direct example of female exogamy. In the case of a 2nd-degree relationship, the type of mobility would be unspecific as the parent leaving La Almoloya could have been either the father or the mother of the female from Lorca (Fig. 3, Supplementary 3).

Using IBD analyses, we also found three 6th–7th-degree inter-site relationships involving pairs of males and female and male individuals from La Almoloya and La Bastida (Dataset S1.7), which connect the networks of biological relatives across both El Argar sites. In addition, we found between-site connections between individuals from phase 3 of La Almoloya and La Horna (LHO), a Valencian BA site ^44,45^. This finding highlights the power to detect long-distance relationships through intensive sampling schemes and emphasizes the extended networks within El Argar, but also sheds light on the political and economic relations with neighboring societies^46^ (Dataset S1.7, Fig. S4)*.*

The combined view of the results leans towards the practice of female exogamy and patrilocality, in which young females moved to a different residence to build new relationships. The adult females buried in double graves provide support for these practices as they have no parents buried at the site and, apart from their offspring, also no other adult relatives, which suggests that they came from outside the community and were integrated through their union with local males, and can thus be considered mates of lineage males. Importantly, the fact that we do not find 1st- or 2nd-degree relationships between adult women at La Almoloya suggests that this practice was reciprocal and that young females from La Almoloya also moved to other sites. Patrilocality does not necessarily imply the absence of mobility of adult males. In fact, our results also support substantial mobility for both sexes as shown by the presence of fewer 2nd-degree than 1st-degree relatives at the site. However, the ability to trace male lineages through generations by the presence of adult male offspring, but not female adult offspring (Fig. 4B), supports patrilocality despite male and female mobility.

The integration of genetic, demographic, and other contextual data sheds further light on the social organization and kinship practices of the Argaric community. Results of the genetic and anthropological sex determination indicate a slight excess of males (11 males vs. 8 females) among subadults. By contrast, the number of adult women (N = 53) exceeds the number of adult men (N = 32), resulting in a sex ratio of 1.65 in favor of females. When separated by chronological phase, we find that the female:male ratio increased over time, from 1.48 (females N = 31; males N = 21) in phase 2 to 2.0 (females N = 22; males N = 11) in phase 3, although this difference is not statistically significant (*X*^2^ = 0.427, df: 1, *p* = 0.513). Most Argaric necropolises have yielded higher numbers of adult females than males ^47^. The opposite trends in infants and adults could be explained by the widely observed sex differences in mortality rates (e.g.,^48,49^), i.e., the loss of males. To this effect, the higher frequency of cranial traumata among Argaric males^50^ raises the possibility that violent conflicts and off-site deaths had resulted in fewer chances for intramural burials, thus leading to the underrepresentation of adult men. However, at the moment, this possibility is only supported by negative evidence. The fact that the number of child burials during phase 3 is three times higher than in phase 2 (30 and 10, respectively) could also point to an increase in child mortality. Both unbalanced factors could be signs of social conflicts, which would be in line with the abandonment of La Almoloya at the end of phase 3 and around the same time as other El Argar sites (El Argar, Gatas, Fuente Álamo or Cabezo Negro) and the subsequent collapse of the El Argar territory border^22,51^.

Alternatively, the unbalanced sex ratios among adults in Argaric cemeteries may account for an ‘excess’ of females rather than a lack of males. This scenario involves not only substantial numbers of incoming women, but also marriage practices consistent with it, i.e., either in the form of polygyny or serial monogamy. Nevertheless, female exogamy alone does not account for all the forms of mobility that could be inferred from the data of La Almoloya. Among men, the lack of evidence for adult brothers and half-brothers suggests that patrilocality might have been restricted to certain males, while others left the site. Men may have moved from their birthplace as part of male exogamy practices, assignment to other settlements, or processes of migration/colonization linked to the Argaric territorial expansion. Future studies on the aDNA of people buried at sites other than La Almoloya will contribute further to the testing of these possibilities. Crucially, marriage practices are not necessarily the only factor conditioning the geographical distribution of people. At La Almoloya, a relatively high number of individuals analyzed (45%) was genetically unrelated beyond the 5th degree. This fact reminds us that economic and political relationships irrespective of biological kinship may strongly influence patterns of mobility throughout space and time, particularly in highly complex societies like El Argar.

### Revisiting the ‘marriage hypothesis’

The reported genetic data from three cases of double tombs of adults suggest that the two adults were mates with common offspring. However, it is not clear that the available data rules out the possibility that other double tombs with adults could also contain more distantly related individuals. Current methods to detect biological relatedness via aDNA data are limited in their capacity to reliably define the precise degrees of relatedness beyond the 4th degree by the quality and preservation of the data, and the completeness of the sampling scheme. However, given sufficient data quality, the detection of relatedness using IBD methods up to the 6–8th degree is as equally reliable and robust as it is for 1st- and 2nd-degree relations, or the detection of “unrelatedness”. This is important to bear in mind when assessing the probability that two adults were genealogically related, but set apart by one century, i.e., approximately three to five generations. Indeed, such cross-generational gaps were suggested by Lull et al. on the basis of a Bayesian analysis of ^14^C date pairs from 23 double tombs from several El Argar sites ^30^. To revisit this hypothesis, we extended the dataset to 38 adult double tombs, for which ^14^C dates for both individuals are available. The results of the re-analysed ^14^C data suggest contemporaneity of both adults in each tomb for the majority of cases (n = 30, 79%), leaving eight tombs (21%) from the previous study from other sites, for which the pairs of ^14^C dates were statistically different (Dataset S1.8). However, as the radiocarbon dates for these tombs were produced in the early days of Accelerator Mass Spectrometry (AMS) dating, systematic re-dating would be needed in order to clarify whether the chronological gap between each pair is real or due to measurement inaccuracies.

On the basis of the new genomic evidence and AMS ^14^C dates in 11 studied cases from La Almoloya, we were able to show that two adults in double burials were unrelated, and certainly not as closely related as the 3rd and 4th degree (considering a generational gap). Additionally, for two double burials with sufficiently high data coverage for IBD-analysis (AY90: ALM020-ALM021, and AY80: ALM015-ALM016) we can safely exclude a genealogical relationship up to the 6th–8th degrees. The coefficient of relatedness between all 11 double burials at La Almoloya is comparable to the vast majority of randomly selected pairs of individuals from BA Iberia. Additionally, several mating pairs are confirmed through their common offspring, lending further support to the idea that at least some mates were buried together. The sequential order of burials by sex can lead to new inferences. In the 19 double tombs, the first individual to be buried was the male in 13 cases (68.4%) and the female on six occasions (31.6%). Thus, from a social perspective, both women and men seem to have been entitled to enter a double tomb first. However, symmetrical rights of females and males do not seem to have extended to mating with multiple partners, because all attested cases of half-siblings were relationships on the side of the father. In any case, if adult double burials emphasized a link between two individuals, then the fact that triple or larger tombs are extremely rare in El Argar would probably render polygyny a marginal practice. Nevertheless, it must be stressed that single, not double tombs, were the usual type of burial for Argaric people. Furthermore, we caution that not every adult, opposite-sex double burial necessarily reflects mating pairs. Archaeologically, the double tombs symbolically express the notion of ‘alliance’ between individuals representing social groups, either through men and women who had offspring together, and/or who partnered in life, but died several decades apart. It appears that the practice of symbolizing such alliances was common during phase 2, but that it became more restricted, both spatially and socially, during phase 3.

### Biological relatedness and social class

Social inequality is another salient feature of the Argaric period. Differences in grave goods, diet, production and access to foodstuff production and metallurgy as well as the means of physical violence (weapons) are striking in the context of EBA Europe. The persistence of such inequalities across centuries calls for hereditary property rights for two of the three social classes identified so far^33^. La Almoloya offers the possibility to trace economic and political differences through lines of descent. However, only limited conclusions can be drawn from such a small number of cases. First, the offspring of intermediate and dominant classes that died at a young age are not common, as most of their diagnostic funerary items were reserved for individuals who died after the age of ~ 15, implying a rite of passage in terms of social age or, rather, the intersection of age and class. Second, the interpretation/attribution of grave goods in double tombs is usually controversial, as it has been observed that some, or perhaps all of the items associated with the first body were removed when the second body was buried. Finally, economic and social privileges and power may not always be transmitted equally and shared by the entire group of close biological relatives.

In the case of La Almoloya, four elite tombs have been documented. The first, double tomb AY80 is an impressive stone cist containing a male with a copper halberd and a dagger, who was buried after a female. We obtained genome-wide data from both individuals, which shows that they were the parents of a man buried in the double tomb AY42. However, in AY42, the only funerary offering was a necklace associated with the second individual in the double tomb, which would imply the loss of the social class over one generation. However, as previously mentioned, grave goods belonging to the first individual in AY42 might have been removed during the second funeral, thereby ‘impoverishing’ it. Double tomb AY60 contains another elite burial, symbolized as a warrior grave, and which we analyzed successfully. Here, the male individual from AY60 (ALM002) was buried after a middle-aged female (ALM001), who was found to be 2nd-degree related to another male (ALM025) from the adult double burial AY94, whose funerary goods are typical of the lower class. This is potentially an example where genetic links do not imply hereditary transmission of wealth and privileges. The remaining halberdier elite burial from phase 2, tomb AY71 could not be analyzed due to poor preservation.

The only elite tomb reported from phase 3 is the lavishly furnished double tomb AY38^22^. According to the genetic results, both individuals lacked clear genealogical links to other adult burials at the site, thus raising the possibility that an ‘external’ ruling elite was involved in drastic architectural changes marking the beginning of phase 3. Intriguingly, the emblematic silver diadem, or ‘crown’, around the woman’s head connects the rulers of La Almoloya with the elites buried at the eponymous El Argar site, 100 km to the south. The adult couple in AY38 were the parents of the first girl buried in AY30 (ALM030). She was the half-sister of the second girl found in the same tomb, whose father is also ALM039 from tomb AY38. Intriguingly, tomb AY30 of the half-siblings is a simple pit without grave goods located in a building next to that of AY38. However, the fine-grained data from stratigraphy and chronology indicate that between the death of the woman ALM038 of AY38 and the deposition of her daughter in AY30 a major social change took place, which entailed the collapse of the palace building in which AY38 was placed. We argue that this could have disrupted power relations and inheritance rules.

### Concluding remarks

The study of biological relatedness at La Almoloya and nearby sites has provided new insights into kinship practices and social organization of the EBA El Argar society. The integration of anthropological, archaeological and genomic results has allowed us to contextualize burial types and results of biological relatedness estimates, such that infant double burials or infant burials in close proximity were found to be siblings or half-siblings. In general, we observe that closer degrees of biological relatedness are reflected in the spatial distribution of the graves at the site.

In contrast to the prevailing hypotheses about Argaric kinship practices, we showed that adult males and females who were co-buried in double tombs were genetically unrelated and thus reflect non-biological kin relations, such as partnerships, marriages or socio-political unions representing human bonds of heterosexual mates who, in three cases, had common offspring buried at the site. This would lend support to the original ‘marriage hypothesis’ as proposed by the discoverers and excavators of El Argar sites^26,27^. However, strictly monogamous, heterosexual couples should not be the only interpretation of double tombs from this time period, as we found one case in which a male (ALM039) from a double burial had offspring with another female.

Moreover, the comparison of the structure of all reconstructed pedigrees suggests practices of patri/virilocality and patrilineality in combination with reciprocal female exogamy. The main support for these kinship practices comes from (a) the absence of maternal parents of adult women buried at the site, the lack of adult daughters of couples buried at the site, and the deficit of 2nd-degree relatives between adult women; (b) reconstructed pedigrees of up to five generations that are linked exclusively through the paternal side, and supported by inferences of distant relatedness through IBD; and (c) cases of half-siblings who shared the same father. The number of cases of half-siblings and chronological resolution do not allow us to distinguish between serial monogamy and polygyny. The attested cases of half-siblings are a clear indication that one individual could have multiple mating partners. However, the absence of triple or multiple burials renders serial monogamy more likely than polygyny, regardless of the reasons for the change of mate (which can vary and not just include untimely death). In addition, we have suggested mobility for males not involved in patrilocal practices. Finally, the substantial number of genetically unrelated individuals calls for political and economic factors most probably embedded in a general framework of alliances and conflicts, in which consanguinity and marriage played just one part.

When considering the chronological developments at La Almoloya, the abundance of double tombs from the earlier phase 2 (2000–1750 cal BCE) appears to emphasize socio-political alliances, as expressed in the funerary rites, among Argaric groups. During phase 3, the relationships within the funerary record appear more inward-oriented, with marked emphasis on close biological lines of descent. Continuous pedigrees spanning both phases render a demographic turnover in phase 3 unlikely, whereas increasing signs of inequality, reflected by the differences in the grave goods, might indicate the effects of shifting regional political rule.

In light of the growing body of intra-site studies from various regions and time periods in prehistoric Europe, we observe a trend towards predominantly virilocal, patrilineal descent groups in combination with higher mobility of females (reciprocal female exogamy), a form of social organization which is already visible during the Neolithic (e.g. Fleury^52^, Hazleton^53^, Newgrange^54^, and Koszyce^55^). Hence, we argue here that the large-scale transformations of the EBA were not necessarily brought about by drastic changes, i.e., violent events and a complete replacement of social structures, but rather that socio-economic and demographic factors met already existing socio-political conditions that furthered the rise of inequality and hierarchization.

Importantly, this study showcases the potential of integrating archeological and genomic evidence in the interpretations of burial rites, kinship practices and social organization at the intra-site level. Future research on comparable sites and regions is needed to draw more nuanced conclusions about the social organization of EBA societies at the pan-European scale.

## Materials and methods

### Sampling strategy

To gain insights into the genetic relationships between individuals buried at La Almoloya we took advantage of the recent extensive excavations and sampled all individuals with suitable morphological preservation (n = 86). We combined these with successfully-typed individuals from other El Argar and Southeastern Iberia BA sites^7,8^, and integrated these results in our discussion (Dataset S1.2).

### Osteological analysis

Osteological analysis has been conducted using Buikstra & Ubelaker standards^56^. Age and sex categories follow the same guidelines. To estimate age at death in subadults we prioritized tooth development (crown formation, eruption and root completion) over degree of epiphyseal closure and diaphyseal length as dental age is more stable and less affected by environmental factors^57^. Adult age at death estimates relied on Suchey & Brooks’ guidelines for changes in the pubic symphysis surface ^58^ and on the morphological changes of the auricular surface of the ilium ^59^. Whenever these criteria could not be used because of conservation problems, age determination of adulthood was based on ectocranial and upper maxillary suture closure and fusion of some ossification centers typical of young adults, as the medial clavicle, the iliac crest, the two first sacral vertebrae or the jugular plate^60^ (Dataset S1.1).

Osteological sex determination was restricted to adults and first relied on pelvic morphology ranked by the presence of any of the three pubic traits originally defined by Phenice (most specially the ventral arc), the shape and breadth of the greater sciatic notch and the presence of a preauricular sulcus^56^. When these criteria could not be used because of poor preservation, sex assessment was based on the five-category spectrum of variation defined by Buikstra and Ubelaker for five cranial traits: nuchal crest protrusion, mastoid process morphology, frontal bone slope, supraorbital margin sharpness and mental eminence outline. It is worthwhile noting that we only observed a single discrepancy between osteological and genetic sex determination: here, a possible male (AY104–ALM029) defined on the basis of a badly preserved skeleton, without pelvic makers and a severely degraded skull, was found to be genetically female (Dataset S1.1).

Finally, double tombs have been studied following standard bioarcheological procedures concerning both cadaveric and skeletal taphonomy in specific funerary contexts. Our observations on body treatment, disposal and sequence of inhumations follow the methodology set by Henri Duday and collaborators in their pioneering works on the postmortem fate of human skeletal remains^61,62^ Dataset S1.1).

### Sample processing and sequencing

Sample preparation, DNA extraction and genomic library preparation were carried out in the aDNA clean room facilities of the Archaeogenetics Departments of the Max Planck Institute for the Science of Human History, Jena, Germany, described in^8^. In total, 153 samples were screened for DNA preservation by shotgun sequencing ~ 5 M Illumina single end reads per partial UDG-treated DNA library^63^ (Dataset S1.2).

### 1240 k SNP capture and sequence data processing

Libraries with an endogenous human DNA > 0.1%, average read length and characteristic aDNA damage patterns > 3% were selected for hybridization capture of 1.24 million informative sites (1240 k SNP panel) on the human genome ^64^. Following standard aDNA processing pipelines ^65^, we quantified contamination rates at the autosomal (in males) ^66^ and mitochondrial level (both genetic sexes) ^67^, showing low contamination estimates (< 3% for nuclear, 5 for mitochondrial DNA). Passing this set of quality criteria, we obtained high quality genome-wide data for 68 individuals from La Almoloya, who allowed genetic kinship analysis (Dataset S1.2).

### Genetic relatedness analysis

We estimated the genetic relatedness between pairs of individuals with more than 1000 shared SNPs using three related methods: PMR^68^ (Dataset S1.4), READ^69^ (Dataset S1.5) and lcMLkin^70^ (Dataset S1.6), and found that the resulting 1st- and 2nd-degree related pairs of individuals were consistent across the three methods (Supplementary 2). However, due to missing data, inherent in ancient DNA studies, we found that only 1st- and 2nd-degree relationships can be ascertained reliably, whereas the error bars for less closely related degrees increasingly overlap. To resolve further degrees of relatedness, we performed IDB analysis on individuals with more than 400,000 SNPs) (Dataset S1.7, Supplementary 2). We integrated the newly generated genomic data as well as the uniparentally-inherited haplogroups, combining all available archeological, chronological and anthropological information, such as relative chronologies based on stratigraphy and the estimated age at death of the individuals (Dataset S1.1, Dataset S1.2). The combined context was also used to infer the temporal directionality of the relationships (Supplementary 3).

## Supplementary Information

Supplementary Information 1.

Supplementary Information 2.

## Data Availability

Genome-wide data have been deposited in the European Nucleotide Archive (ENA) under accession number PRJEB46907.
