# Supplementary material for: Kinship practices in the early state El Argar society from Bronze Age Iberia
Source: Sci Rep. 2022 Dec 27;12:22415. doi: 10.1038/s41598-022-25975-9 (PMC9794729; doi:10.1038/s41598-022-25975-9)
Supplement: Supplementary file 2 — Supplementary Information 2. [file 41598_2022_25975_MOESM2_ESM.pdf]

Supplementary materials

S1 Archaeological background

The site of La Almoloya (Pliego, Murcia), ca. 3,100 m^2 in size, is a relatively small El Argar settlement, located in the northern foothills of Sierra Espuña. However, in terms of preservation of architecture and volume of means of production it is comparable to the eponymous site of El Argar, which was excavated in the 19th century, but where hardly anything but ca. 1,000 burials were documented[1]. While large El Argar settlements usually expand over terraced hill slopes, La Almoloya is a more or less flat setting on a natural, boat-shaped plateau protected by vertical escarpments. The site lies ca. 35 km NE from La Bastida, another El Argar urban settlement (4.5 ha), where nearly 100 tombs have been explored in our recent excavations. While La Bastida is protected in the mountains, La Almoloya (585 m a.s.l.) has an exceptional visual control over the lowlands and valleys of Murcia, and a view stretching even to southern Alicante.

According to available ^{14}C dates, La Almoloya was continuously occupied between 2,200 and 1,550 cal BCE, spanning the entire El Argar period. The architectural and stratigraphic sequence differentiates three settlement phases, further divided into subphases. During the early and middle phases, La Almoloya seems to have been an important border settlement from which the northward expansion of El Argar might have been organized, as the unusually high proportion of warrior graves containing halberds suggests. During the final phase (1750-1550 cal BCE), La Almoloya not only developed into an urban site but also seems to have become a palatial center of the El Argar ruling class. The plateau was occupied by at least nine building aggregates, separated by narrow passages or drainage channels. The three largest buildings, with a constructed area ranging between 266-332 m^2, were placed in the central-western part of the settlement. Each building contained 6-10 rooms on the ground floor, though some living and working spaces continued on a second level. Several rooms were used as specialized storage areas and workshops, including large-scale cereal processing, textile manufacture, grinding stone production and resharpening, wood- and fiber working, honey and wax processing, etc. Apart from a water cistern in the north-eastern corner of Building Complex 3, the most outstanding architectural feature is Room 9 in Building Complex 1 with continuous stone benches along all four walls, where over 50 persons could sit, and a large fireplace (2 m^2) in front of an altar/throne-like platform. The scarcity of archeological findings, especially tools, supports the idea that this 70 m^2 room was used as a space of political gathering.

Under one of the benches, one of the richest tombs (AY38) of the EBA in Europe was discovered[2]. Between 2013 and 2016, 101 intact burials were excavated at La Almoloya, 46 of which were from phase 2 of the settlement (ca. 2000-1750 BCE) and 55 from phase 3 (ca. 1750-1550 BCE) (four cenotaphs that date to phase 3 have not been included). Spatially, they are scattered over the whole of the excavation area, though not all rooms contain tombs beneath the occupation layer, nor do all tombs correspond to documented habitation areas. In fact, most of the architecture of phase 2 was dismantled during the construction of the urban plan of phase 3, at around 1750 BCE.

S2 Estimating biological relatedness

We used several methods to estimate the degree of biological relatedness among individuals based on genome-wide sequence data from all the Iberian BA individuals available [3–5]. We applied PMR (a method to calculate the pairwise mismatch rate)[6] and READ [7] to our pseudo-haploid 1240k SNP dataset. Both methods rely on the number of mismatches among 1240k SNP positions in individual pairs to estimate the coefficient of relatedness. We also used LcMLkin[8], a method which uses genotype likelihoods, to estimate the probability that at any site in the genome, two individuals share zero (k_0), one (k_1) or two (k_2) alleles that are identical by descent. Finally, for individuals with more than 500k SNPs covered we used GLIMPSE (https://github.com/odelaneau/GLIMPSE) to phase and impute genotype calls, applying the default parameters (https://odelaneau.github.io/GLIMPSE/tutorial_hg19.html)[9] and using the 1000 genomes reference panel[10]. Samples with more than 600k SNPs with the genotype posterior of >= 0.99 after imputation were included in the downstream IBD analysis. We used ancIBD (https://pypi.org/project/ancIBD/) to call and summarize identity-by-descent (IBD) blocks of 8, 12, 16 and 20 cM size shared between pairs of individuals.

S2.1 Baseline of unrelated pairs Iberian BA individuals and coefficient of relatedness

We calculated the PMR for all pairs of BA individuals from Iberia[3–5]. We excluded individuals whose genotypes were restricted to damaged reads (labeled in the dataset as “_d”), as they tend to accumulate more transitions (C to T and G to A mutations) that artificially inflate the PMR, resulting in 12,720 pairs from 160 individuals (**Fig. S1A, Dataset S4**). In **Fig S1** we plot the PMR values against the number of shared SNP

positions for each pair of individuals. We calculated a median PMR value of 0.25541 after excluding pairs who shared less than 2,000 SNPs, and we established this value as the baseline for unrelated pairs (**Fig. S1, Dataset S1.4**).

We also calculated the median PMR value including only pairs of individuals with more than 10,000 shared SNPs, following[11] but the value was the same to the 3rd decimal place.

After showing that none of the individuals from[5] indicated lower PMR values when compared with published individuals[3,4], which excludes any close biological relationship (1st- or 2nd-degree) between different datasets/sites, we created a subset of BA individuals from[5], which were geographically and temporally most relevant to our study. From this data set we were able to calculate a more accurate regional baseline value for unrelated pairs (**Fig. S1B, Dataset S1.4**) resulting in a median value of 0.25424. From this subset, we had excluded individuals, which did not have at least one reliable contamination estimate (BAS007, BAS006, FAL007, ALM033, ALM037, ALM045 and CBR001). We plotted, but did not include ZAP002 in the baseline calculation, as this individual was identified as an outlier[5] and thus yielded higher PMR values (**Fig. S1B, Dataset S1.4**). Values below the median likely represent individuals who are more closely related. To calculate the coefficient of relatedness of the individuals showing values below the median of the population we used the following equation for the individuals *i* and *j*, denoted

$$cij = pU- pijpT,$$

where pij is the pairwise-mismatch rate for individuals *i* and *j*, pU is the expected PMR for two unrelated individuals (found as the median of all of the pij), and pT is the expected PMR for identical individuals (calculated as pU/2).
A coefficient of relatedness (x) of ~1 indicates the same individual or identical twins, ~0.5 1st-degree related and ~0.25 2nd-degree related individuals.

On the basis of the PMR, we estimated a coefficient of relatedness ranging from 0.458543-0.538468 for 1st-degree relatives, and a coefficient of relatedness ranging from 0.222467-0.298773 for 2nd-degree relatives. The pair ALM034-MMI003 yielded a coefficient of relatedness that falls between the 1st and 2nd degree (0.419053) (**Fig. S1B, Dataset S1.4**).

S2.2 Estimating the degree of biological relatedness using READ

We used the software READ (Relationship Estimation from Ancient DNA)[7] to estimate biological relatedness up to the 2nd degree in pseudo-haploid low coverage data from the 1240k SNP panel (**Fig S2**, **Dataset S1.5**). This method calculates the proportion of non-matching alleles in windows of 1 Mbps for each pair of individuals (p_0). p_0 is normalized using the median of all average pairwise p_0 across all pairs of individuals, which, if the sample size is sufficient, will be a reliable estimate of the expected p_0 for a pair of unrelated individuals[7]. Depending on the normalized proportion of shared alleles, the software classifies all pair comparisons among individuals as the best fitting degree of relationship: unrelated, 2nd-degree related, 1st-degree related or identical individuals/identical twins. The uncertainty of the fitted assigned degree of relatedness is reflected in the reported upper and lower Z-scores, applying a cut-off of |2|.

We ran READ on the population subset of El Argar individuals published in[5] and were able to replicate the results obtained via PMR (**Fig S1B**). **Fig S2** shows the results of the first 70 pairwise comparisons from READ and **Dataset S1.5** lists the results of all pairwise comparisons. In **Fig S1A** and **Fig S1B** we highlighted the individual pairs (red outline) classified as 1st- or 2nd-degree related with a non-significant upper Z-score. These pairs have to be interpreted with caution and the degree of relatedness requires further support from either uni-parentally inherited markers, the position in the pedigree with respect to other individuals, and the chronostratigraphy and archaeological context. Individual pairs classified as either 1st- or 2nd-degree related, but with no significant upper and lower Z-scores due to low coverage, were excluded from the plot (**Fig S2**).

S2.3 Estimation of biological relatedness using LcMLkin.

We also applied LcMLkin[8] to pairs of individuals with > 1,000 shared SNPs. We ran the data using two different thinning parameters (–thin 50,000 and 10,000), with the results shown in **Fig. S3** and reported in **Dataset S1.6**. The 1st- and 2nd-degree related pairs determined using LcMLkin confirm the results obtained by PMR and READ (**Fig. S1** and **Fig. S2**). Based on the results of the LcMLkin analysis, we observe three pairs of individuals with a k_0 value that would be more consistent with a sibling-sibling relationship than a parent-child relationship, however the archeological context and how each pair is related to other individuals renders a parent-offspring relationship more plausible. We note that these three pairs also share less than 5,000 overlapping SNPs, and, as a

consequence, the k_0 values are likely downwardly biased. These pairs of 1st-degree relatives are ALM015-ALM052, ALM030-ALM038 and ALM030-ALM039, and the description of the reconstructed pedigree together with the archaeological details are explained below (**Supplementary S3**).

S2.4 Estimation of biological relatedness using IBD

Identity-by-descent (IBD) blocks are identical haplotype segments shared by two individuals. The overall length of these is expected to decrease with each recombination event, i.e. during each meiosis. As a result, the normalized total number and sum of the length categories can provide insights on the degree of relatedness between two individuals. We called shared IBD blocks of >8, >12, >16, and >20 cM in individuals with more than 600k SNPs after imputation with GLIMPSE (https://github.com/odelaneau/GLIMPSE) using ancIBD (https://pypi.org/project/ancIBD/). On the basis of contrasting the inferred total number and length of IBD blocks in a bidimensional plot (**Fig. S4**) we can establish the degree of relatedness between pairs of individuals from 1st to 6-7th degree, based on the principle of counting the number of meioses for each degree of relatedness (e.g., aunt-nephew are 2nd degree related but only one meiosis had happened, whereas a 2nd degree grandparent-grandchild pair underwent two meioses).

S3 Reconstructing pedigrees at La Almoloya

S3.1 Pedigrees involving 1st-degree relationships

A. AY21-1/ALM073 and AY21-2/ALM062 (Fig. S5A)

Individuals AY21-1/ALM073, a 30-35-year-old woman, and AY21-2/ALM062, a female neonate, were 1st-degree relatives, most likely mother-daughter. A sibling relationship is highly unlikely as the archaeological and anthropological context strongly suggest a simultaneous double burial, and the skeletons were found partly intertwined. A scenario of two sisters buried in this position and an age gap of at least 30 years between siblings appears less plausible. This pair of individuals had less than 1,000 shared SNPs and was analyzed with only PMR and READ. Both individuals carried mtDNA-haplogroup H1j and have been dated to phase 2 of the site (2000-1750 cal BCE) (**Fig. S5A**). READ produced

a Z-score of 2.287 for the pair being 2nd degree, whereas the archaeological context and results from the uniparentally-inherited markers support a 1st-degree relationship.

B. AY17/ALM077, AY8/ALM068 and AY23/ALM078 (Fig. S5B)

Individuals AY17/ALM077 (an adult female) and AY8/ALM068 (an infant female) were 1st-degree relatives. In addition, AY23/ALM078 (an infant male) was related in the 2nd degree to AY8/ALM068 but unrelated to AY17/ALM077. The stratigraphic context suggests that AY23/ALM078 was buried last. We thus infer that AY8/ALM068 and AY23/ALM078 could have been half-siblings on their father's side. We cannot rule out the possibility that AY23/ALM078 was an uncle of AY8/ALM068, i.e. the younger brother of her father, and buried after her. As both half-siblings and the uncle/niece scenarios are relationships through the male line, this accounts for why no 1st-, 2nd- or 3rd-degree relationship was observed between AY23/ALM078 and AY17/ALM077.

As a consequence, individuals AY17/ALM077 (adult female) and AY8/ALM068 (infant female) were most likely mother and daughter. If instead we assumed that they were full siblings, both of them would be expected to be 2nd-degree related to AY23/ALM078, which was not the case.

Finally, the mother (AY17/ALM077) and daughter (AY8/ALM068) carried the same mtDNA haplotype, whereas the half-siblings did not. Based on stratigraphy we infer that AY17/ALM077 was interred before her daughter (AY8/ALM068). The three individuals come from the third phase of El Argar, ca. 1750-1550 cal BCE (**Fig. S5B**).

C. AY-88/ALM080 and AY89/ALM081 (Fig. S5C)

We found AY-88/ALM080 and AY89/ALM081 to be 1st-degree relatives. Only a sibling relationship is possible because both were infants (Infans I at the time of death), which rules out a parent/offspring relationship. Both individuals were genetically male and carried the same mt-haplogroup (U5b3) and Y-chromosome haplogroup (R1b-P312) consistent with a sibling relationship on both uni-parentally inherited lines. The two individuals were buried in single graves less than 2m apart and under the same housing complex (H27) of phase 3 (**Fig. S5C**). First-degree relationships were confirmed by all methods used and full sibling relationships were confirmed by IBD (**Fig. S4, Dataset S1.7**).

D. AY26-2/ALM086, AY27-1/ALM058, AY50/ALM064 and AY75/ALM014 (**Fig. S5D**)

We found that individuals AY26-2/ALM086 (adult female) and AY27-1/ALM058 (adult male) were 1st-degree relatives, and that AY27-1/ALM058 was also related in the 1st degree to individual AY50/ALM064 (adult male), which is confirmed by the observed 2nd-degree relationship between AY26-2/ALM086 and AY50/ALM064. AY26-2/ALM086 and AY27-1/ALM058 share the mtDNA haplotype (U5b1f1), different from the mtDNA-haplotype carried by AY50/ALM064 (H1cf). This scenario is consistent with AY26-2/ALM086 being the mother of AY27-1/ALM058 and the paternal grandmother of AY50/ALM064.

We also detected a 2nd-degree relationship between individuals AY75/ALM014 and AY27-1/ALM058, and our data is consistent with a 3rd-degree relationship between AY-75/ALM014 and AY50/ALM064. This scenario can be explained if AY-75/ALM014 was the paternal uncle of AY27-1/ALM058, and carries a different mtDNA-haplotype, which indicates a relationship on the father's side.

We can rule out a sibling relationship between AY26-2/ALM086 and AY27-1/ALM058, because in that case we would expect no close biological relationship between AY27-1/ALM058 and AY75/ALM014. We can also exclude a sibling relationship between AY27-1/ALM058 and AY50/ALM064, because they do not share the same mtDNA-haplotype and AY75/ALM014 is consistent with a 3rd-degree relationship with AY50/ALM064, but not with a 2nd-degree relationship, as observed between AY75/ALM014 and AY27-1/ALM058.

The chronological sequence of the funerary record based on radiocarbon dates and stratigraphic context is consistent with the reconstructed pedigree: AY26-2/ALM086 and AY75/ALM014 are dated to phase 2, while AY50/ALM064 is stratigraphically dated to phase 3, highlighting the existence of close biological connections between archaeological phases 2 and 3 (**Fig. S5D**).

Lastly, we were able to establish a grandmother/grandson relationship between AY26-2/ALM086 and AY50/ALM064 through IBD analysis (**Fig. S4, Dataset S1.7**). READ results yielded significant Z-scores for all 1st- and 2nd-degree relationships (**Fig. S3, Dataset S1.5**).

F. AY22-1/ALM048, AY22-2/ALM049 and AY16/ALM034 (**Fig. 3**)

Individuals AY22-1/ALM048 and AY22-2/ALM049 were not biologically related but both were related in the 1st degree to AY16/ALM034. When two unrelated individuals are equally related in the 1st degree to a third individual, this must mean that they had the third individual as offspring together. In this case, AY22-2/ALM049 (father) and AY16/ALM034 (son) were genetically determined to be males and AY22-1/ALM048 (mother) a female. AY22-1/ALM048 (mother) and AY16/ALM034 (son) share the same mt-haplogroup (K1a and K1a+195 respectively). Likewise, AY22-2/ALM049 carries Y-chromosome haplogroup R1b-P310 (P312 is not covered due to low resolution, and P310 is thus consistent with P312) and AY16/ALM034 R1b-Z195, consistent with a parent/offspring relationship to the limits of our resolution. All three adult individuals were buried close to each other. The unrelated male and female (AY22-1/ALM048 and AY22-2/ALM049) were located in a highly disturbed double burial where both skeletons were fully disarticulated but rearranged in a way that replicates the layout that is typical of double tombs at La Almoloya. Their son's burial, located 2 m to the south, had also been disturbed by the later building activities of phase 3 (**Fig. 3**). The 1st-degree relationship between AY16/ALM034 and AY22-2/ALM049 and AY22-1/ALM048 was confirmed by PMR, READ (significant Z_upper and Z_lower), and LcMLkin. Using these methods, we found a close relationship between AY16/ALM034 and MMI003, a 40-50-year-old female from Lorca, buried in Madres Mercedarias tomb 4, another Argaric site about 50 km away. READ results supported a 1st-degree relationship with a non-significant Z_upper (0.433), while the coefficient of relatedness from the PMR calculation was more compatible with being between 1st-2nd degree. If AY16/ALM034 and MMI003 were indeed related in the 1st degree, we should expected to find a 2nd-degree relationship between MMI003 and the parents of AY16/ALM034. A 2nd-degree relationship between MMI003 and AY22-1/ALM048 could be confirmed by READ, but the Z_upper was also non-significant (Z_upper = 0.390; Z_lower = -2.022) and thus not fully reliable. Likewise, READ results from the analogous pair MMI003 and AY22-2/ALM049, also returned non-significant Z-scores, and thus render the result inconclusive (rather than being unrelated). The lack of power of resolution is due to the low coverage of individual MMI003. The radiocarbon dates for AY16/ALM034 and MMI003 do not allow to distinguish between 1st and 2nd degree relatedness. Therefore, although there are hints of a close relationship between AY16/ALM034 and MMI003, these results need to be taken with caution.

G. AY38-1/ALM038, AY38-2/ALM039, AY30-1/ALM030 and AY30-2/ALM031 (Fig. 3)

The two individuals buried in the double tomb AY38, AY38/1-ALM038 (an adult female) and AY38-2/ALM039 (an adult male) are not closely related but both are 1st-degree relatives of the individual AY30-1/ALM030 (a 14-17-month-old girl). As outlined above, the only possibility for this group is a trio of mother/father/child.

In addition to this core trio, we have found a 2nd-degree relationship between AY30-1/ALM030 and AY30-2/ALM031, another double tomb also from phase 3 but located 5 m to the north under a different housing complex. As AY30-2/ALM031 is unrelated to AY38-1/ALM038 but a 1st-degree relative of AY38-2/ALM039, we interpreted AY30-1/ALM030 and AY30-2/ALM031 as half-siblings on the father's side (AY38-2/ALM039). The only alternative way of accounting for the 2nd-degree relationship, and - at the same time - the 1st-degree relationship between AY30-1/ALM030 and AY38-2/ALM039 would be to assume that AY30-2/ALM031 is the sister of AY38-2/ALM039 (and thus paternal aunt of AY30-1/ALM030). However, we can exclude this scenario because AY30-2/ALM031 and AY38-2/ALM039 do not share the same mtDNA haplogroup (**Fig. 3**). Significant Z-scores from READ support a 1st-degree relationship between ALM038-ALM030 (Z-scores 4.47, -9.81), ALM039-ALM030 (Z-scores 7.03, -11.89) and ALM039-ALM031 (Z-scores 5.43, -20.13). The pair ALM030-ALM031 yielded a non-significant Z_Upper value of 1.694, whereas the archaeological context and results from uniparentally-inherited markers support the half-sibling relationship between ALM030-ALM031.

The READ results suggested a 2nd-degree relationship (Z_upper = 2.571) between CMO002, a middle-aged adult male from a double burial (Cerro del Morrón tomb 1) found at the border of El Argar territory, 60 km to the north of La Almoloya, and the female baby ALM030. However, we could not confirm a closer relationship also between CMO002 and ALM038 or ALM039, the parents of ALM030. Since the girl died prematurely (14-17 months), a continuation of the pedigree from her line is impossible. As a result, a 2nd-degree relationship is not supported. Nevertheless, a close biological relationship (>2nd-degree) between ALM030 and CMO002 cannot be excluded.

H. AY80-1/ALM015, AY80-2/ALM016, AY42-1/ALM052, AY80-0/ALM017 and AY28/ALM060 (Fig. 3)

Individuals from the double burial AY80-1/ALM015 and AY80-2/ALM016 were both related in the 1st degree to individual AY42-1/ALM052 but were themselves unrelated, which is

consistent with a scenario in which they are the biological parents (mother and father) of AY42-1/ALM052. In addition, AY80-0/ALM017 (remains of the skull of an adult male deposited just outside the tomb where AY80-1/ALM015 and AY80-2/ALM016 were buried) was a 1st-degree relative of AY28/ALM060 (an infant female). Since they do not share the same mtDNA-haplotype, a sibling relationship can be ruled out and, therefore, a father/daughter relationship is the only plausible interpretation.
At the same time, using PMR we observe a close biological connection between the individuals AY80-0/ALM017 and AY80-2/ALM016 which could be explained as a 3rd-degree relationship. Additional IBD analysis confirmed that AY80-1/ALM015 and AY80-0/ALM017 were great-grandmother and great-grandson, respectively, and also indicated a 4th-degree relationship between AY80-1/ALM015 and AY28/ALM060. READ results yielded statistically significant support for all of the 1st-degree relationships of the pedigree. Together, all of the results can be connected along the paternal line to a larger pedigree of five generations, which span the two archaeological phases at the site. At the top of the pedigree is the double burial of AY80-1/ALM015 and AY80-2/ALM016 and the chronologically youngest representative of this lineage is a great-great-granddaughter, AY28/ALM060 (**Fig. 3**).

S3.2 Description of 2nd- and 3rd-degree relatives not involved in any 1st-degree connected pedigree

Individual AY082-1/ALM018 (35-40-year-old female, mtDNA-haplogroup R0a) was related in the 2nd degree to AY097-2/ALM028 (adult male, mtDNA-haplogroup K1a+195). This suggests a grandparent/grandchild, uncle-aunt/nephew-niece or half-sibling relationship on the father's side, as they do not share the same mtDNA-haplotype. Both adults were assigned to El Argar phase 2.
Individual AY058/ALM004 (30-35-year-old female, mtDNA-haplogroup U5a1) was related in the 2nd degree to AY057/ALM075 (4-5-year-old female, mtDNA-haplogroup U5b1e). This implies a grandmother/granddaughter, aunt/niece or half-sibling relationship on the father's side, as they do not share the mtDNA-haplotype. The upper Z-score from READ was not significant (Z-score = 1.44), but the results from the PMR and LcMLkin were consistent.
Individual AY087/ALM019 (30-40-year-old female, mtDNA-haplogroup K1b1a1c), who is one of the oldest burials of La Almoloya, and AY095/ALM069 (~3-year-old male, mtDNA-

haplogroup H1q1) were found to be 2nd-degree related. Both carried different mtDNA-haplotypes, which can only be explained as a grandmother/grandchild, aunt/nephew or half-sibling relationship on the paternal side. Also here, the upper Z-score from READ was not significant (Z-score = 0.83), but the results from the PMR and LcMLkin were consistent.

Individuals AY013/ALM046 (a 12-14-month-old male, mtDNA-haplogroup T2+16189) and AY014/ALM047 (6 or 7-month-old male, mtDNA-haplogroup H6a1b) were 2nd-degree related. Both individuals were buried in the same building and were assigned to the same archaeological phase. Based on the age at death of both children, we are able to rule out a grandfather-grandson relationship, and being buried in the same context and the same archaeological phase makes a half-sibling scenario more plausible than an uncle-nephew relationship. The different mtDNA haplotypes allow us to infer a half-sibling relationship on the paternal side as the most likely scenario.

Individual AY060-1/ALM001 (40-49-year-old female, mtDNA-haplotype X2b+226) was a 2nd-degree relative of AY094-2/ALM025 (30-40-year-old male, mtDNA-haplotype H3). The different mtDNA haplotypes indicate a grandparent/grandchild, uncle-aunt/nephew-niece or half-sibling relationship on the father's side. Both adults come from the same archaeological phase. We detected an additional 2nd-degree relationship between individuals AY094-2/ALM025 and AY090-1/ALM020 (35-40-year-old male, mtDNA-haplotype K1a3a), again on the paternal side, but we could not resolve the genealogy further. It is noteworthy that we also found a relationship of a higher degree (potentially the 3rd) between ALM020 and ALM001 based on the PMR value. One possibility would be that ALM025 was the grandfather of ALM020 and ALM001, who are then cousins on the father’s side. The upper Z-score from READ for the pair ALM001-ALM025 was 2.02, but results were consistent with the PMR and LcMLkin.

Finally, a 2nd-degree relationship was inferred for individuals AY82-2/ALM045 and AY97-2/ALM028. This relationship has not been included in the general count due to the lack of contamination estimation for the individual ALM045 (low coverage). The upper Z-score from READ was not significant (Z-score = 0.03) and the coefficient of relatedness calculated via the PMR results was 0.2, which would be within the range of a 2nd degree pair.

S4 Pedigrees from nearby Bronze Age sites

MDP001-MDP003

The two adult male individuals from the Molinos de Papel BA site were found to be 1st-degree related. Both males carried different mtDNA-haplogroups (MDP001 is K1a and MDP003 is HV0), but highly likely the same Y-chromosomal haplogroup (R1b-M269 and R1b-P312, within the limits of our resolution), which is consistent with a father/son relationship, or vice versa. Individual MDP003 was buried in a double grave with adult female MDP002, who is unrelated to both adult males, MDP001 and MDP003. Only in a scenario where MDP001 is the son of MDP003, would the scenario be similar to at La Almoloya, where an unrelated pair of adults represent mates, and the offspring of the father with another woman who has not been analyzed.

LHO001-LHO002

The two male infants from the BA site La Horna were also found to be 1st-degree related. IBD results indicate that they were full siblings, which is consistent with the age at death of the boys and both carrying the same mtDNA-haplogroup K1a+195 and the same Y-chromosomal Y-haplogroup R1b-Z195, and the age at death of the boys.

BAS017-BAS018 and BAS003

READ yielded results suggesting a 1st-degree relationship between the 9-11-month-old female baby BAS017 and the 9-11-month-old male baby BAS018 from the Argaric site of La Bastida, although the upper Z-score was not significant (Z-score = 1.30). Since both individuals were of the same age at death, buried together in grave BA23, and also carried the same mtDNA-haplogroup U5b1, a full sibling relationship between BAS017 and BAS018 is the most parsimonious interpretation. Intriguingly, we found that the adult female BAS003 was also suggested to be 1st-degree related to BAS017, with an upper Z-score of 2.41. However, since the two individuals carry different mtDNA haplogroups (BAS003 carries H1), which is impossible for a 1st-degree relationship between females, and in the absence of high-quality genome-wide data that would permit IBD-analysis, we assume a more distal relationship between BAS003 and BAS017 on the paternal side.

S5 Radiocarbon dating

S5.1 Testing the contemporaneity of double tombs

Double tombs are a characteristic feature of El Argar intramural burial rites. Anthropological and taphonomic observations confirm that both individuals were buried successively, but cannot inform on the time lapse between these two events. In order to assess the contemporaneity of these individuals, we radiocarbon dated 11 double burials from La Almoloya and one from Cerro del Morrón (**Fig S6**). All pairs of dates are statistically undifferentiated at 95% (calculations performed with Calib 8.2 - http://calib.org/calib/; option 'Sample Test Significance')[12].

S5.2 Refining the chronological phases of La Almoloya

We generated eighteen new radiocarbon dates that were added to the previously published dataset from La Almoloya burials, reporting a total of 43 radiocarbon dates. We applied two-phase Bayesian modeling as implemented in OxCal v4.4[13], without constraining values with the objective of refining the chronological boundaries of each archaeological phase at La Almoloya and the transition time from one phase to another, assuming that they are continuous as we observed from our connected pedigrees. In addition, we implemented the Kernel plot function in order to determine the density distribution of the events in each archaeological phase[14] (**Fig. S7**).
The two-phase Bayesian model yielded an index of agreement of 98.3 (A_{model}=98.3%). The Individual agreement index was higher than 60%, a cut-off that is considered acceptable for the model [13] in all of the samples (ranging from 111.7 to 76.8%) except for AY87/ALM019 (49% of agreement) and AY82-2/ALM045 (46% of agreement). The radiocarbon range for those individuals exceeded the beginning of phase 2 **(Fig. S7**), but from the genetic analysis it was clear that AY87/ALM019 (adult female) was 2nd-degree related to AY95/ALM069 (infant male), which suggests that there could only have been a maximum of one generation between the two individuals. We also found that AY82-2/ALM045 (adult male, 46% of agreement) was 2nd-degree related to AY97-2/ALM028 (adult male, agreement of 106%). These two results indicate that the chronological date of AY87/ALM019 and AY82-2/ALM045 was likely at the younger end of the distribution curve, which falls into the range of dates associated with phase 2.
Applying the two-phase Bayesian model to the new radiocarbon dates confirms the narrow boundaries between archaeological phases, consistent with the observation of pedigrees

that span the phases despite the attested architectural restructuring of the settlement at La Almoloya at the time[15]. This transition thus implies more social and/or economic changes than biological ones as genetic continuity was observed at population level[5] as well as pedigrees at an individual level.

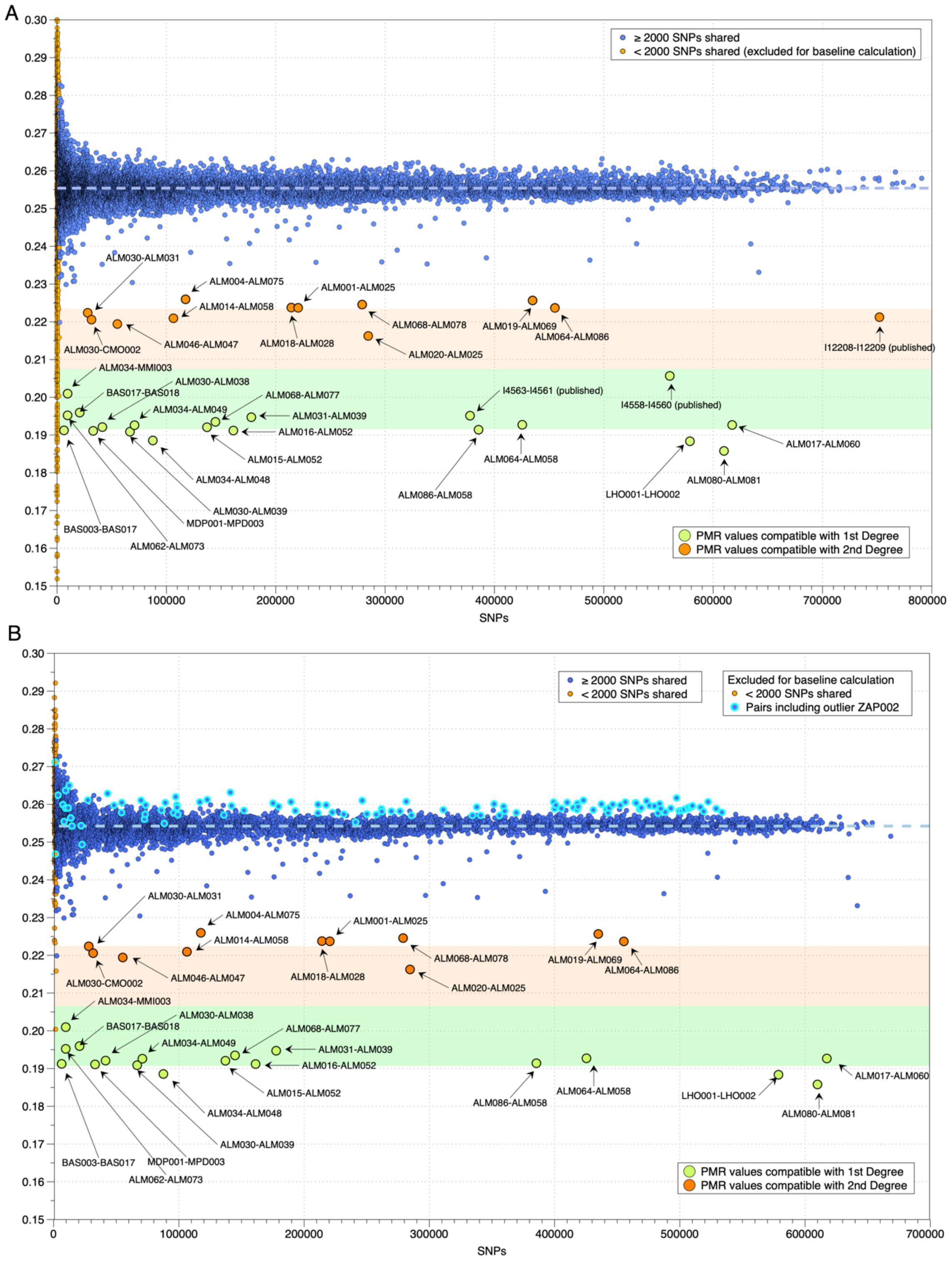

Fig. S1. First- and 2nd-degree related pairs of individuals obtained using PMR. **(A)** baseline calculation for unrelated pairs using the Iberian BA dataset; **(B)** baseline calculation for unrelated pairs using the El Argar and South Eastern Iberia BA dataset published in[5].

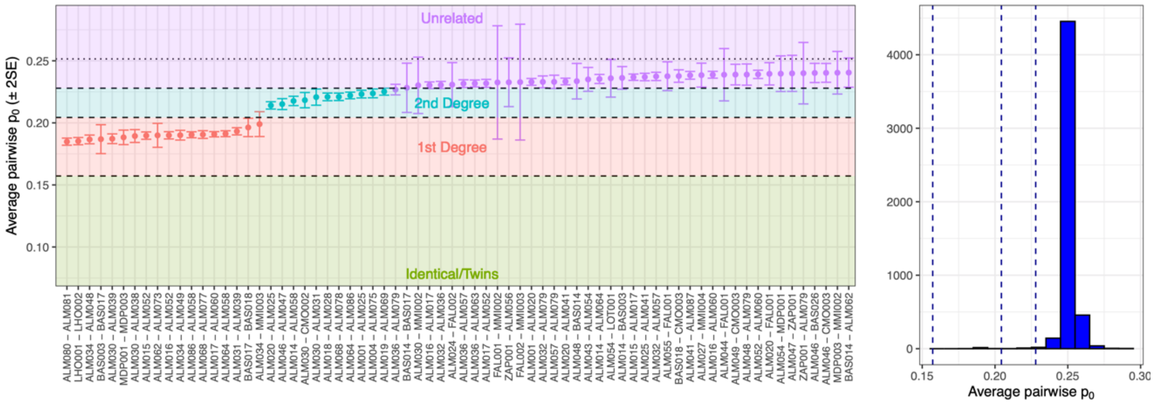

Fig. S2. READ results. Ordered, median Pairwise mismatch rate (PMR) obtained from READ for the first 70 individuals. First- (blue) and 2nd-degree (red) related pairs yielded significant upper Z-score bounds.

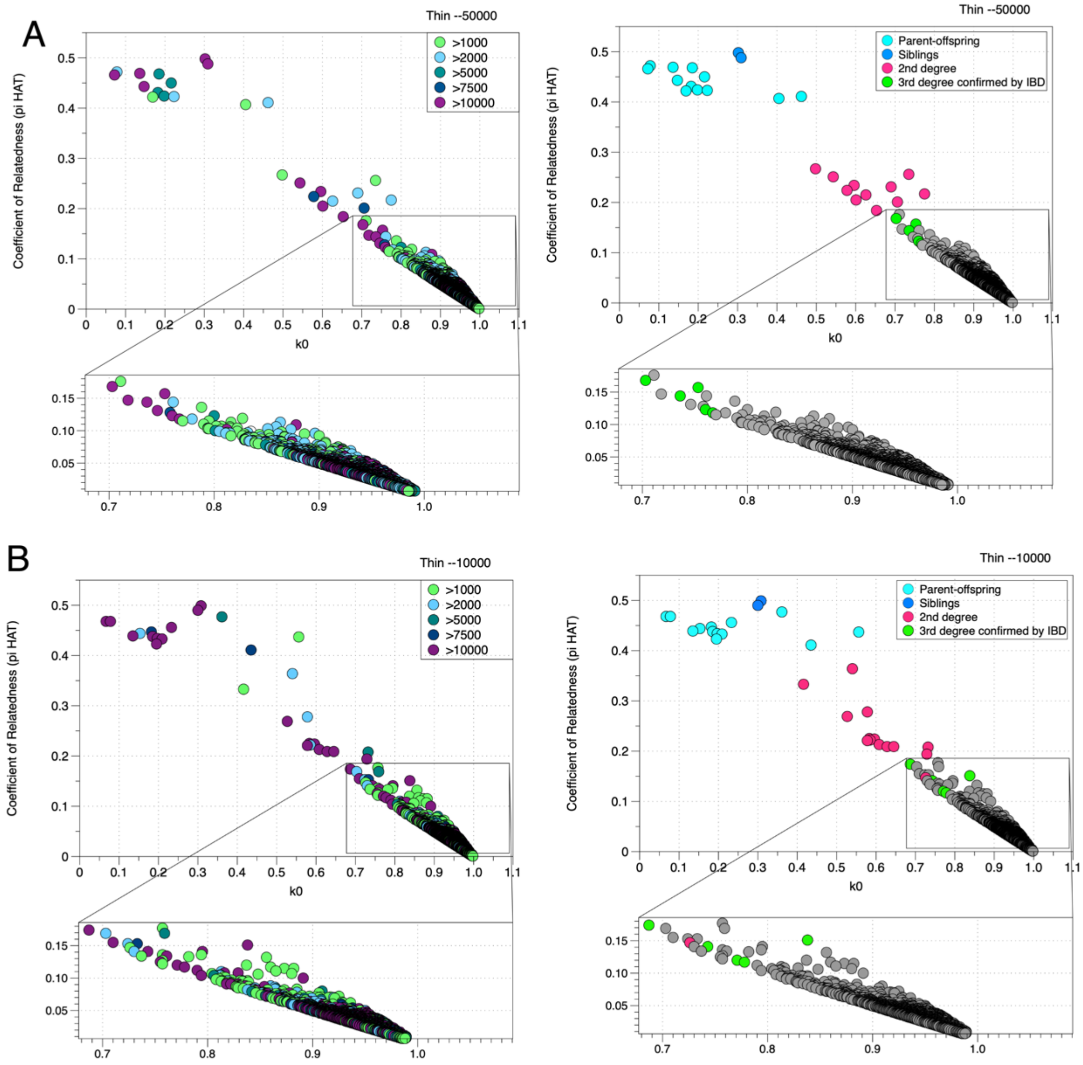

Fig. S3. LcLMkin results for pairs of individuals with at least 1,000 shared SNPs using different thinning parameters. **(A)** The Coefficient of Relatedness versus k_0 calculated with LcMLkin (colors indicate SNP coverage) (left); and The Coefficient of Relatedness versus k_0, highlighting 1st (blue) and 2nd (red) degree pairs of individuals (right) using the parameter *--thin 50000*. **(B)** The Coefficient of Relatedness versus k_0 calculated with LcMLkin (colors indicate SNP coverage) (left); and The Coefficient of Relatedness versus k_0, highlighting 1st- (blue) and 2nd-degree (red) pairs of individuals using the parameter *--thin 10000* (right).

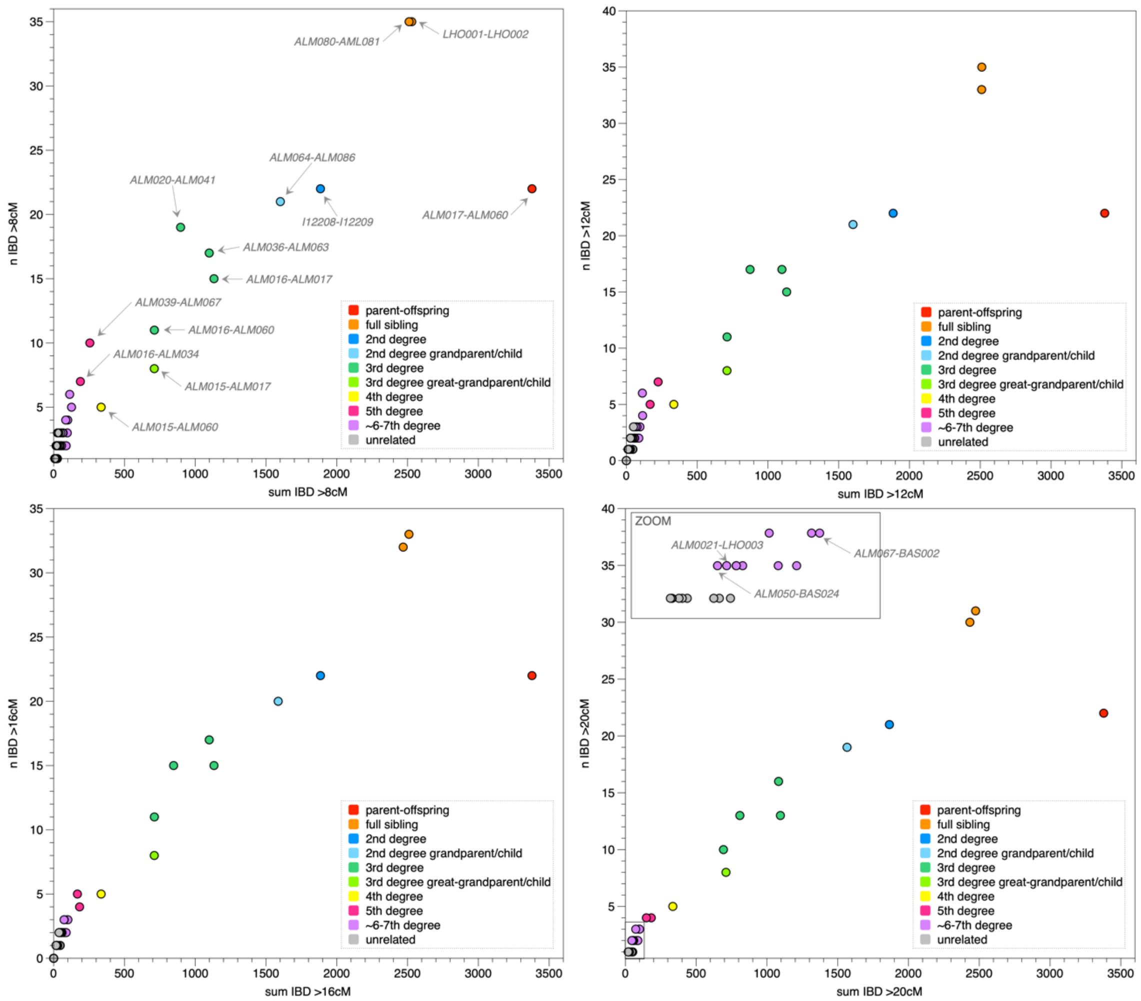

Fig. S4. Shared IBD blocks of various sizes >8cM, >12cM, >16cM and >20cM between pairs of individuals with more than 600k SNPs after imputation (**Dataset S1.7**).

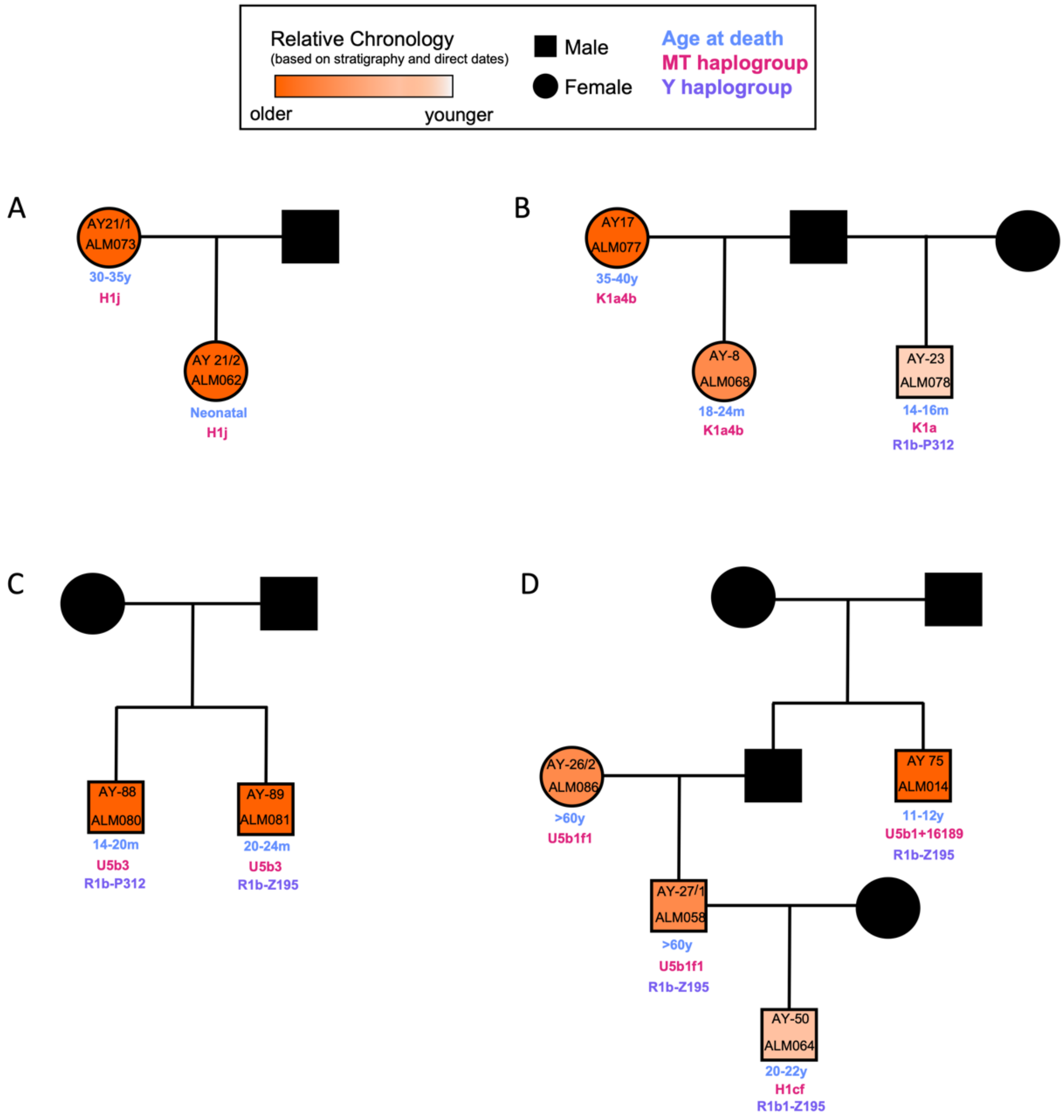

Fig. S5. Reconstructed pedigrees involving all 1st- and 2nd-degree relationships observed at La Almoloya. Details of each pedigree in panel A-D and **Figure 3** are described in the text.

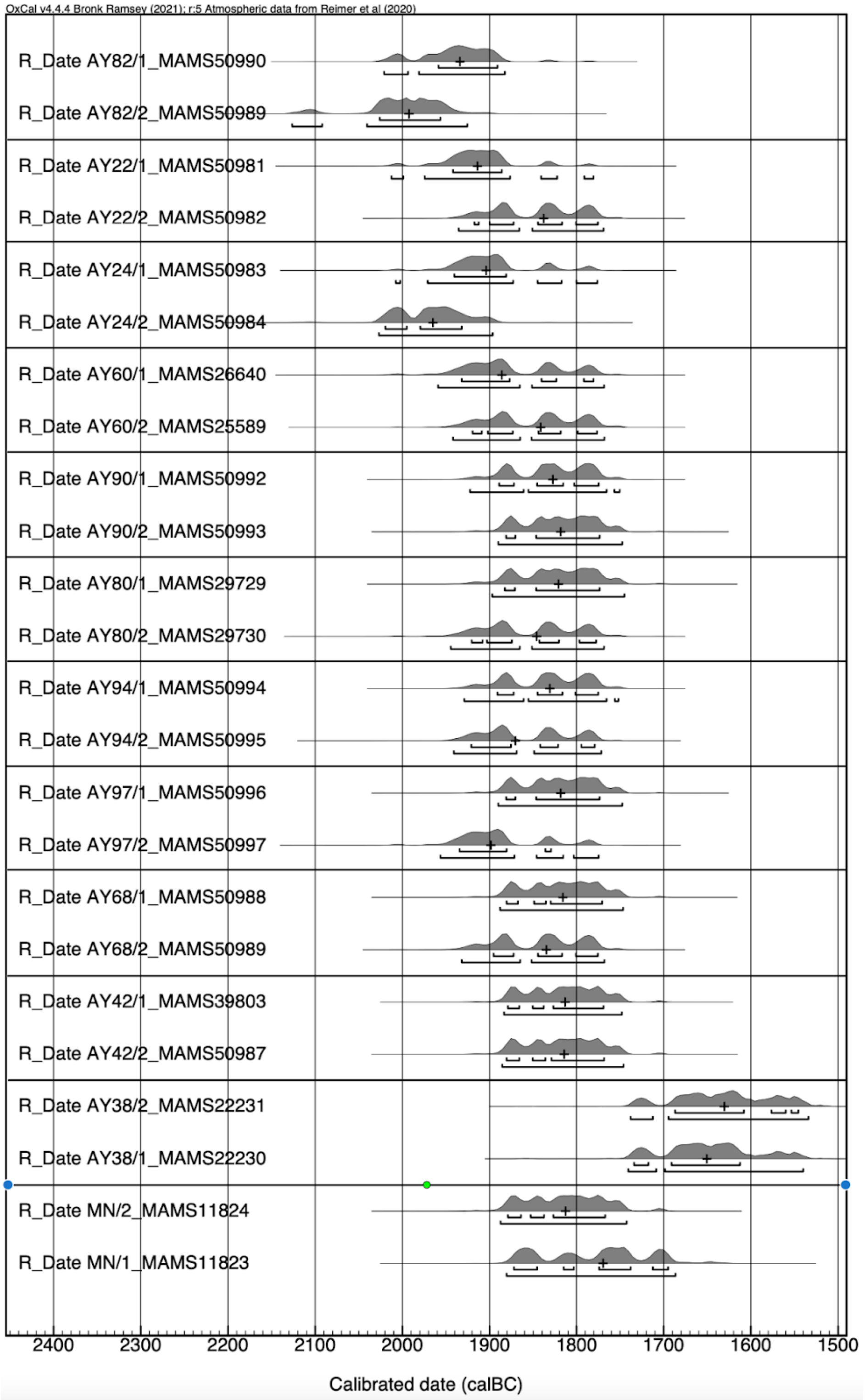

Fig. S6. Calibrated radiocarbon dates for the adult individuals buried in the double tombs with DNA results from La Almoloya (AY) and Cerro del Morrón (MN). AY82 only yielded DNA results for individual AY82/1. For each pair of dates, the first one belongs to the

individual buried in the first place but in the case of Cerro del Morrón where no depositional data are available.

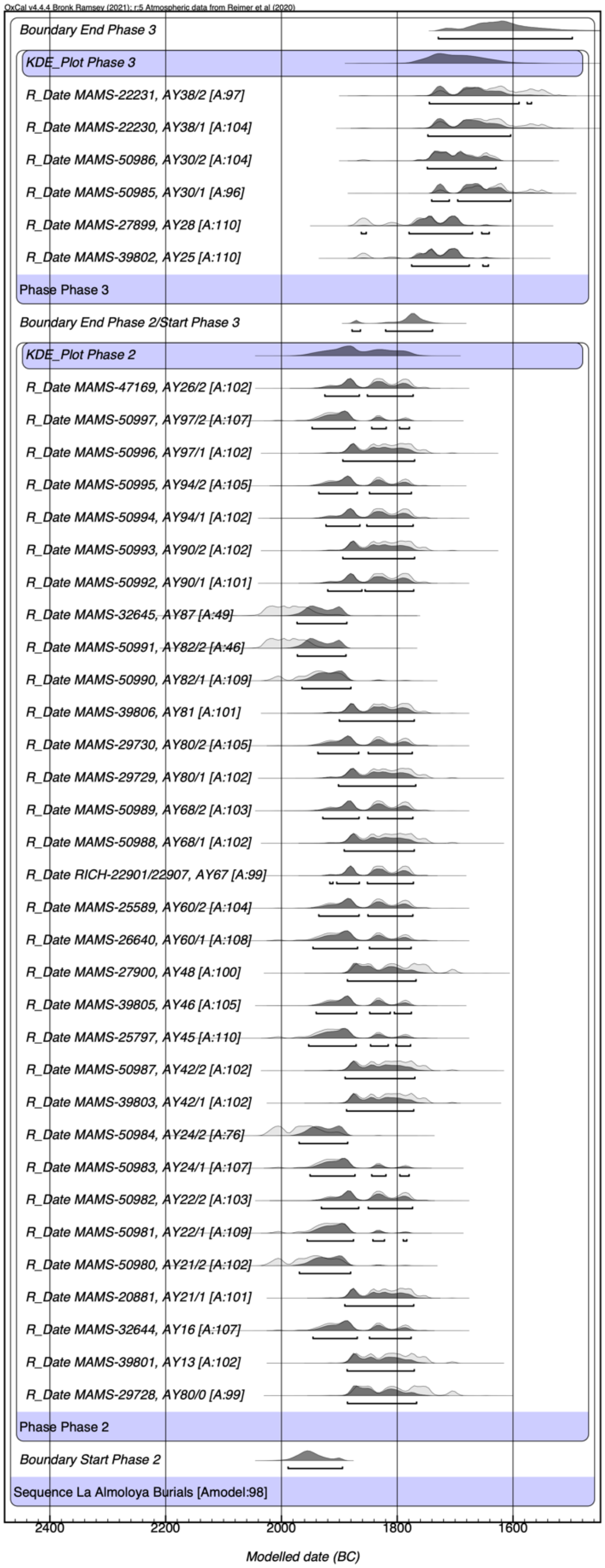
OxCal v4.4.4 Bronk Ramsey (2021); r:5 Atmospheric data from Reimer et al (2020)
Boundary End Phase 3
KDE_Plot Phase 3
R_Date MAMS-22231, AY38/2 [A:97]
R_Date MAMS-22230, AY38/1 [A:104]
R_Date MAMS-50986, AY30/2 [A:104]
R_Date MAMS-50985, AY30/1 [A:96]
R_Date MAMS-27899, AY28 [A:110]
R_Date MAMS-39802, AY25 [A:110]
Phase Phase 3
Boundary End Phase 2/Start Phase 3
KDE_Plot Phase 2
R_Date MAMS-47169, AY26/2 [A:102]
R_Date MAMS-50997, AY97/2 [A:107]
R_Date MAMS-50996, AY97/1 [A:102]
R_Date MAMS-50995, AY94/2 [A:105]
R_Date MAMS-50994, AY94/1 [A:102]
R_Date MAMS-50993, AY90/2 [A:102]
R_Date MAMS-50992, AY90/1 [A:101]
R_Date MAMS-32645, AY87 [A:49]
R_Date MAMS-50991, AY82/2 [A:46]
R_Date MAMS-50990, AY82/1 [A:109]
R_Date MAMS-39806, AY81 [A:101]
R_Date MAMS-29730, AY80/2 [A:105]
R_Date MAMS-29729, AY80/1 [A:102]
R_Date MAMS-50989, AY68/2 [A:103]
R_Date MAMS-50988, AY68/1 [A:102]
R_Date RICH-22901/22907, AY67 [A:99]
R_Date MAMS-25589, AY60/2 [A:104]
R_Date MAMS-26640, AY60/1 [A:108]
R_Date MAMS-27900, AY48 [A:100]
R_Date MAMS-39805, AY46 [A:105]
R_Date MAMS-25797, AY45 [A:110]
R_Date MAMS-50987, AY42/2 [A:102]
R_Date MAMS-39803, AY42/1 [A:102]
R_Date MAMS-50984, AY24/2 [A:76]
R_Date MAMS-50983, AY24/1 [A:107]
R_Date MAMS-50982, AY22/2 [A:103]
R_Date MAMS-50981, AY22/1 [A:109]
R_Date MAMS-50980, AY21/2 [A:102]
R_Date MAMS-20881, AY21/1 [A:101]
R_Date MAMS-32644, AY16 [A:107]
R_Date MAMS-39801, AY13 [A:102]
R_Date MAMS-29728, AY80/0 [A:99]
Phase Phase 2
Boundary Start Phase 2
Sequence La Almoloya Burials [Amodel:98]
2400
2200
2000
1800
1600
Modelled date (BC)

Fig. S7. Bayesian model of contiguous phases for La Almoloya burials based on ^{14}C dates from human bone with OxCal[13] and using the IntCal20 calibration curve[16].

Dataset S1: Detailed archaeological data and genomic data analysis

S1.1 Anthropological and burial description of the individuals analyzed from La Almoloya.

S1.2 Genetic description of the individuals analyzed and their corresponding anthropological and chronological data.

S1.3 Comparative frequencies of the full skeletal sample from La Almoloya and individuals with positive aDNA results from the same site, grouped by age, sex and relative chronology.

S1.4 Coefficient of relatedness calculated via PMR for all Iberian BA individuals and for La Almoloya and other relevant BA sites with the boundaries established for 1st and 2nd degree of relatedness.

S1.5 Degree of relatedness calculated via READ[7] software for La Almoloya and other relevant BA sites.

S1.6 Degree of relatedness calculated via LcMLkin[8] software for La Almoloya and other relevant BA sites.

S1.7 Degree of relatedness calculated via ancIBD (https://pypi.org/project/ancIBD/) for La Almoloya and other relevant BA sites.

S1.8 Adult double burials radiocarbon dates calibrated with IntCal20[16]. All calculations were performed with OxCal 4.4[13].

Supplementary References

1. Siret, E. & Siret, L. *Les Premiers Ages du Metal dans le Sud-Est de l'Espagne*. (Antwerp, 1887).
2. Lull, V. *et al.* Emblems of power and spaces of power during the Argaric Bronze Age in La Almoloya (Murcia, Spain).*Antiquity* (2020).
3. Valdiosera, C. *et al.* Four millennia of Iberian biomolecular prehistory illustrate the impact of prehistoric migrations at the far end of Eurasia. *Proceedings of the National Academy of Sciences* (2018).
4. Olalde, I. *et al.* The genomic history of the Iberian Peninsula over the past 8000 years. *Science* **363**, 1230 LP–1234 (2019).
5. Villalba-Mouco, V. *et al.* Genomic transformation and social organization during the Copper Age–Bronze Age transition in southern Iberia. *Science Advances* **7**, eabi7038 (2021).
6. Kennett, D. J. *et al.* Archaeogenomic evidence reveals prehistoric matrilineal dynasty. *Nat. Commun.* **8**, 14115 (2017).
7. Monroy Kuhn, J. M., Jakobsson, M. & Günther, T. Estimating genetic kin relationships in prehistoric populations. *PLoS One* **13**, e0195491 (2018).
8. Lipatov, M., Sanjeev, K., Patro, R. & Veeramah, K. R. Maximum Likelihood Estimation of Biological Relatedness from Low Coverage Sequencing Data. *bioRxiv* 23374 (2015).
9. Rubinacci, S., Ribeiro, D. M., Hofmeister, R. J. & Delaneau, O. Efficient phasing and imputation of low-coverage sequencing data using large reference panels. *Nat. Genet.* **53**, 120–126 (2021).
10. 1000 Genomes Project Consortium *et al.* A global reference for human genetic variation. *Nature* **526**, 68–74 (2015).

11. Mittnik, A. *et al.* Kinship-based social inequality in Bronze Age Europe. *Science* **366**, 731–734 (2019).
12. Stuiver, M., Reimer, P. J. & Reimer, R. W. CALIB 8.2 [WWW Program]. 2021. (2020).
13. Ramsey, C. B. Bayesian Analysis of Radiocarbon Dates. *Radiocarbon* **51**, 337–360 (2009).
14. Ramsey, C. B. Methods for Summarizing Radiocarbon Datasets. *Radiocarbon* **59**, 1809–1833 (2017).
15. Lull, V. *et al. La Almoloya (Pliego-Murcia): Murcia: Integral, sociedad para el desarrollo rural*. (2015).
16. Reimer, P. J. *et al.* The IntCal20 Northern Hemisphere Radiocarbon Age Calibration Curve (0–55 cal kBP). *Radiocarbon* **62**, 725–757 (2020).
